# Association between respiratory hospital admissions and air quality in Portugal: A count time series approach

**DOI:** 10.1371/journal.pone.0253455

**Published:** 2021-07-09

**Authors:** Ana Martins, Manuel Scotto, Ricardo Deus, Alexandra Monteiro, Sónia Gouveia

**Affiliations:** 1 Institute of Electronics and Informatics Engineering of Aveiro (IEETA) and Department of Electronics, Telecommunications and Informatics (DETI), University of Aveiro, Aveiro, Portugal; 2 Center for Computational and Stochastic Mathematics (CEMAT), Department of Mathematics, IST, University of Lisbon, Lisbon, Portugal; 3 Instituto Português do Mar e da Atmosfera, I.P. (IPMA, I.P.), Lisbon, Portugal; 4 CESAM, Department of Environment and Planning, University of Aveiro, Aveiro, Portugal; 5 Center for R&D in Mathematics and Applications (CIDMA), University of Aveiro, Aveiro, Portugal; Institute for Advanced Sustainability Studies, GERMANY

## Abstract

Although regulatory improvements for air quality in the European Union have been made, air pollution is still a pressing problem and, its impact on health, both mortality and morbidity, is a topic of intense research nowadays. The main goal of this work is to assess the impact of the exposure to air pollutants on the number of daily hospital admissions due to respiratory causes in 58 spatial locations of Portugal mainland, during the period 2005-2017. To this end, INteger Generalised AutoRegressive Conditional Heteroskedastic (INGARCH)-based models are extensively used. This family of models has proven to be very useful in the analysis of serially dependent count data. Such models include information on the past history of the time series, as well as the effect of external covariates. In particular, daily hospitalisation counts, air quality and temperature data are endowed within INGARCH models of optimal orders, where the automatic inclusion of the most significant covariates is carried out through a new block-forward procedure. The INGARCH approach is adequate to model the outcome variable (respiratory hospital admissions) and the covariates, which advocates for the use of count time series approaches in this setting. Results show that the past history of the count process carries very relevant information and that temperature is the most determinant covariate, among the analysed, for daily hospital respiratory admissions. It is important to stress that, despite the small variability explained by air quality, all models include on average, approximately two air pollutants covariates besides temperature. Further analysis shows that the one-step-ahead forecasts distributions are well separated into two clusters: one cluster includes locations exclusively in the Lisbon area (exhibiting higher number of one-step-ahead hospital admissions forecasts), while the other contains the remaining locations. This results highlights that special attention must be given to air quality in Lisbon metropolitan area in order to decrease the number of hospital admissions.

## Introduction

Despite legal and regulatory improvements, particularly in European Union, air pollution remains a pressing problem worldwide. Recent evidence shows that there is an annual excess of nearly 800 thousand deaths due to air pollution in Europe alone [1]. Moreover, it is estimated that air pollution reduces the mean life expectancy in Europe by about 2.2 years [1] on average. A recent study on 652 cities [2] concluded that, on average, an increase of 10*μg*/*m*^3^ in the 2-day moving average of PM_10_ (Particulate Matter with aerodynamic diameter less than or equal to 10*μm*) concentration, is associated with an increase of 0.44% and 0.47% in all-cause mortality and respiratory mortality, respectively. For PM_2.5_ (Particulate Matter that have aerodynamic diameter less than or equal to 2.5*μm*) the increase in mortality is 0.68% for all-cause mortality and 0.74% for respiratory mortality.

The International Agency for Research on Cancer (IARC) classified air pollution and PM mixture as carcinogenic, with evidence of increased risk of cancer even at levels below the current World Health Organization (WHO) PM_2.5_ guideline [3, 4]. However, current legal limits of this air pollutant in Europe are generally above the recommended by the WHO [5]. Hence, it is of the utmost importance to assess the impact of the current levels of air pollution on populations’ health. Yet, in some countries, including Portugal, research on the effect of air pollution on health, either on mortality or morbidity (i.e., hospital admissions), has been scarce [6–10]. The few studies performed have found some associations between health outcomes and air quality, even though, results are not consistent throughout studies. For instance, Alves *et. al* (2010) did not find a significant association between PM_10_ and hospital admissions, whereas Cruz *et*. al (2016) found a significant association between the pollutants PM_10_/PM_2.5_ and respiratory diseases for ages below 15 years. The recent study by Franco *et. al* (2020), restricted to the Lisbon metropolitan area, used ordinary least regression and found significant associations between several air pollutants (PM_10_, NO_2_, NO, O_3_, CO) and respiratory hospital admissions [10]. Notwithstanding, even tough these research studies are somewhat recent, the data used are 15–20 years old and may not reflect the current impact of air pollution on health. Furthermore, the data is restricted to Lisbon area, which may not accurately represent the reality of other country regions less urbanised and populated, and calls for a study nationwide study in Portugal.

The assessment of the effect of air pollution on respiratory hospital admissions demands the inclusion of the temperature effect, since its impact on health outcomes is well-known [11] and temperature has been shown to be associated with some air pollutants [12–14], being their interaction a possible mechanism to explain health outcomes [15]. Therefore, the overall goal of this research work is to quantify the influence of air pollution on respiratory morbidity, beyond the effect of temperature, in Portugal mainland, using as proxy respiratory hospital admissions’. Moreover, for each location the one-step-ahead forecast distribution is estimated and subsequently used in a cluster analysis, which attempts to establish spatial and temporal hospital admission patterns across the country.

It is worth to mention that, arguably, the most common methodology used in the literature to assess the effect of air pollution on health are Generalised Additive Models (GAM) [16]. In GAMs, the response time series is modelled as a linear combination of smooth functions, in general, cubic splines. The use of splines allows the modelling of long-term patterns and, can also capture the seasonal pattern of the data [17]. However, splines have the inconvenient of needing that the number of knots, which governs how many (cubic) curves will be used, are previously established by the researcher [17]. Furthermore, in this particular context, their use increases the mathematically complexity and reduces interpretability [17]. In contrast to the well-established GAM framework, the interest in time series models to deal with discrete outcomes (i.e., counts) has being gaining attention recently [18]. One example of such models are the INteger Generalised AutoRegressive Conditional Heteroskedastic (INGARCH) models which exhibit an ARMA-like structure, although the data generating mechanism is analogous to that of a GARCH model in the sense that, the conditional mean recursively depends on past conditional means and on past observations [19, 20]. The INGARCH formulation incorporates link/transformation functions [21], to deal with negative serial correlation [22] and, time-varying covariates [23, 24]. Moreover, the INGARCH class is able to capture seasonality and serial dependence through the regression on past observations and the autoregression on past conditional means. Hence, unlike GAMs, these models do not require the non-parametric transformation of the predictor variables, resulting in simpler models. As a consequence, model interpretability is straight-forward and comprehensible.

In this work, the INGARCH model with time-dependent covariates is considered for modelling purposes. Note that, the construction of such models requires optimal criteria for covariate selection. The importance of such criteria is obvious as model performance can be improved by ignoring irrelevant covariates and incorporating only relevant covariates at different lags within models’ structure. Indeed, the need for a systematic approach to lag selection has long been identified [25]. Such criteria should also address collinearity, as a strong association among covariates may obscure their relationship with the response, and may lead to computational instability in model estimation. Thus, this paper introduces a novel method for optimal selection of time-varying covariates which will be referred to as block-forward (BF). Briefly, blocks of colinear covariates are considered in a hierarchical order (according to the degree of evidence/impact on health of air pollutants) and, from each block, only the covariate leading to the lowest Akaike Information Criteria (AIC) model is included. Also, such covariate is introduced in the model if and only if all others remain significant. Having in mind that different lagged versions of a single covariate can be thought as colinear covariates, the BF method allows to systematically select the optimal lag for a given covariate. The advantage of such approach relies on the fact that it enables to articulate the empirical knowledge of the effect of air pollution on health as well as statistical criteria so that models can correspond to a more accurate representation of reality.

Hence, the contribution of this work is two-fold; first, an exhaustive analysis of the impact of air pollution on hospital admissions due to respiratory causes, beyond the effect of temperature, in Portugal mainland is performed along with a cluster analysis to identify patterns within the data set. Secondly, a methodological contribution to deal with covariates selection based on empirical knowledge and statistical criteria is introduced.

The rest of the article is unfolded as follows: Section 2 presents a detailed description of the data set and of the methodology used to perform the statistical analysis. Results and discussion are included in Section 3. Finally, Section 4 is devoted to conclusions.

## Materials and methods

### Exploring the data sets

**Fig 1:**
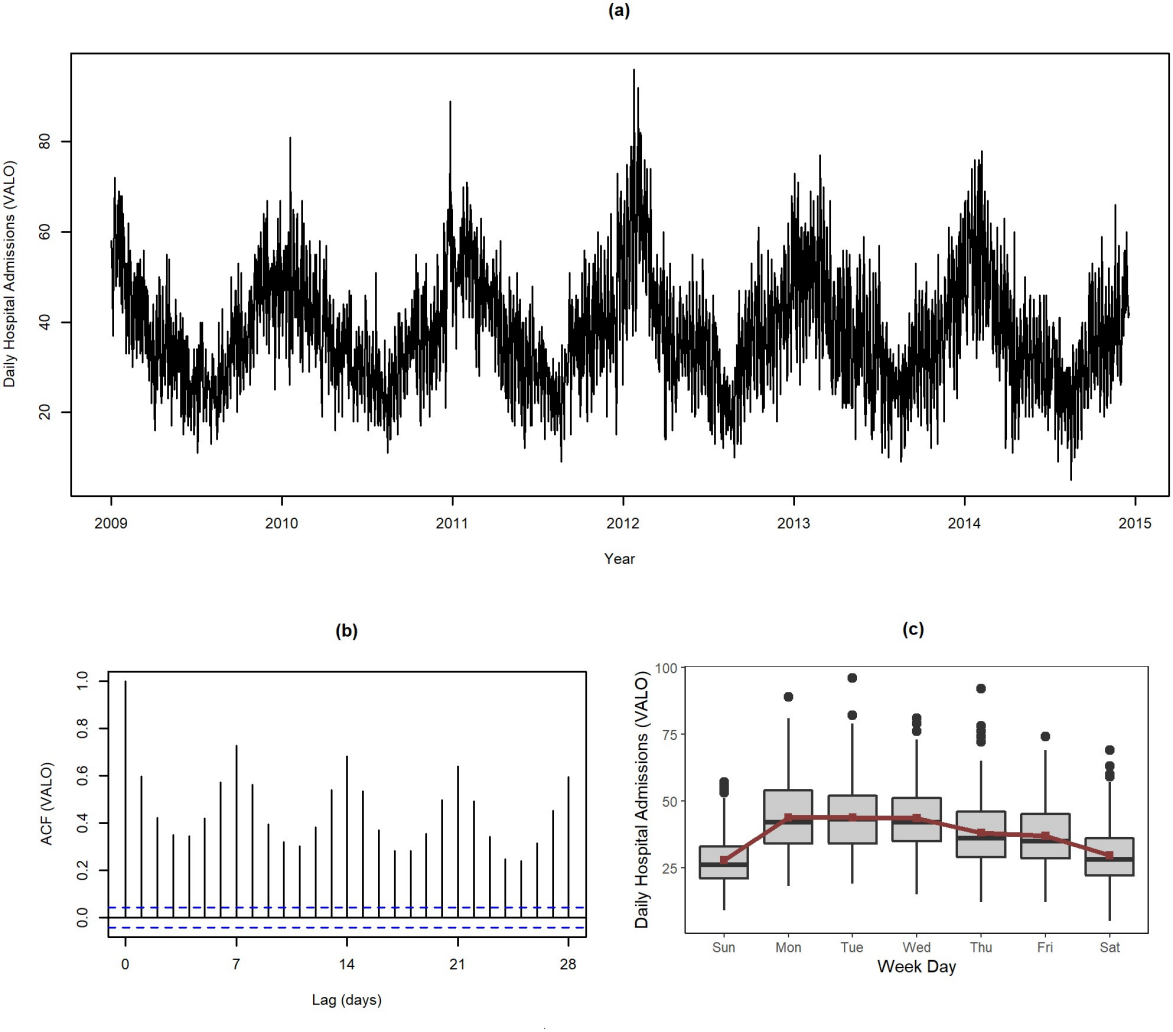
Number of daily hospital admissions at Valongo (VALO) from 2009 to 2015. **(a)** time series, **(b)** sample ACF and **(c)** distribution (box plots) and averaged values (red color dots and line) per day of the week.

Anonymised data from the Homogeneous Diagnostic Groups (HDG) containing data on hospital admissions episodes between 2005 and 2017 was provided by Administração Central do Sistema de Saúde (ACSS). For each spatial location, the time series of the daily number of hospital admissions due to respiratory causes was recorded as the count of episodes resulting from respiratory system diseases’ (ICD-9 codes 460–519 and ICD-10 codes J00-J99) on a daily basis. Fig 1(a) shows the daily number of hospital admissions in Valongo (VALO), Porto District (Portugal), which will be used for illustrative purposes throughout the article. Clearly, the time series exhibits an annual seasonal pattern showing increasing counts from the late summer and during the colder months of the year (with a peak around February) followed by a downward pattern until around August. The time series also displays a weekly periodic pattern as highlighted in Fig 1(b), by the periodic sample ACF pattern with more pronounced values at the multiple of 7-day-lags. Furthermore Fig 1(c), shows a downward trend in the counts from Monday to Sunday. Weekend admissions also differ from weekdays, with the number of hospital admissions being lower at weekends, as suggested by a recent systematic review based on 68 studies covering over 640 million worldwide hospital admissions [26].

**Fig 2:**
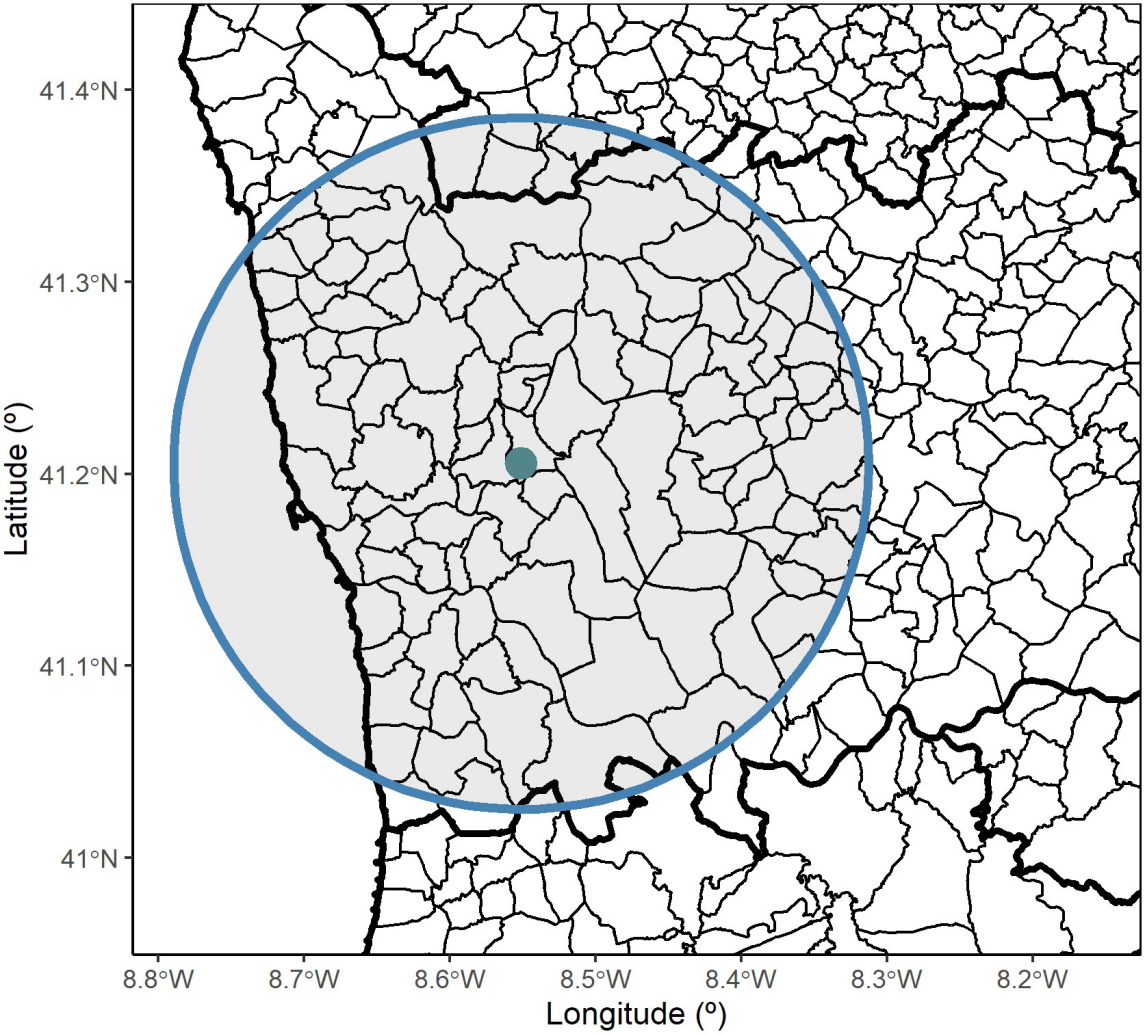
Radius of influence with 20km radius and centered at Valongo (VALO) air quality monitoring station. The polygons identify parishes.

The daily hospital admissions and air quality data were paired considering an influence circumference defined around each monitoring station, illustrated in Fig 2 for Valongo. The corresponding time series of daily hospital admissions was produced as the daily number of episodes associated with residents within the influence area. In this analysis, the radius of 20km was preferred over other radius (e.g. 10, 15, 25 km) by assuming that the air quality indicators measured in each of the 58 monitoring locations are spatially representative for a circle within a 20km radius. It is worthwhile to point out that 20km is much lower than the representative area set in a radius of 100km, roughly corresponding to the life-time of NO_2_ and the formation of secondary particles [27, 28]. Moreover, 20km corresponds to the minimum radius allowing for a sufficient number of counts (hospital admissions) for modelling purposes and thus, it constitutes a fair choice to assess the association between the exposure and outcome.

Using ArcGis (version 10.3.1) the code for each parish within every influence area was retrieved using the codes from the administrative division previous to 2013. It is worth to mention that in 2013 the administrative divisions at parish level were re-organised, which lead to a decrease of the number of parishes. New codes were added to the newly reformulated administrative division and a matching between ‘old’ and ‘new’ codes was performed to accommodate all parishes from 2013 onward. Using the residence code associated to the data set provided by ACSS it was possible to link to the administrative division. Therefore, it was possible to retrieve all cases occurring within each influence area. Note that some parishes are on the limit of the influence area so, to avoid including cases of underrepresented parishes, only those with an area of at least 10% were included in the influence area. This decision does not considerably change the number of cases. Cases were ordered according to date of occurrence and, events happening on the same date were added to obtain the cumulative cases per day, resulting in the daily hospital admissions time series at each influence area.

Air quality data in Portugal is publicly available at QualAr website (www.qualar.apambiente.pt). Hourly data for air pollutants PM_2.5_, PM_10_, NO_x_, NO_2_, CO, O_3_ and SO_2_ were retrieved from all available monitoring stations within the period 2005–2017. Aiming at the analysis of daily hospital admissions, daily time series of air pollutants were computed from the (hourly) available air quality data. In accordance with governmental recommendations [29], maximum daily values were computed by retrieving the maximum daily value when at least 75% of daily observations (i.e., 18 observations) were available at a given day, otherwise a missing value was obtained. Whenever required, missing data is imputed using the k-nearest neighbours (k-NN) method with *k* = 1. Specifically, missing data were replaced by the daily value of the nearest neighbour, i.e. the time series exhibiting the most similar temporal behaviour according to the Heterogeneous Euclidean-Overlap Metric (HEOM) [30], instead of the closest geographically time series. This procedure allows to preserve the mean and standard deviation of the original time series in the imputed data [31]. An air pollutant at a given spatial location was considered in the subsequent analysis provided that the corresponding time series has at least 5-years of consecutive data. In the 58 Portuguese monitoring stations, a total of 58 time series of NO_x_/NO_2_, 54 of PM_10_, 45 of O_3_, 36 of SO_2_, 26 of CO and 18 time series of PM_2.5_ were considered in the analysis. The characterisation of each monitoring station (geographical coordinates, type of environment and influence) as well as the time period analysed is summarised in S1 Table.

Finally, temperature (°C) at 1.5 meters is collected by the Instituto Português do Mar e da Atmosfera, IPMA (www.ipma.pt/pt/index.html), at over 100 locations across the territory. Overall, 27 stations were selected based on their proximity to air quality stations and data availability from 2005 onward. Temperature was provided by IPMA as maximum daily time series. Likewise, 1-NN was used for missing data imputation. It is worth to mention that the effect of temperature on hospital admissions is well-established [32] and thus, temperature is expected to be largely included in models. In this scenario, the models will allow to assess the impact of air quality on respiratory hospital admissions beyond that of temperature. Descriptive statistics of the time series included in this research work can be found in S2 and S3 Tables.

**Fig 3:**
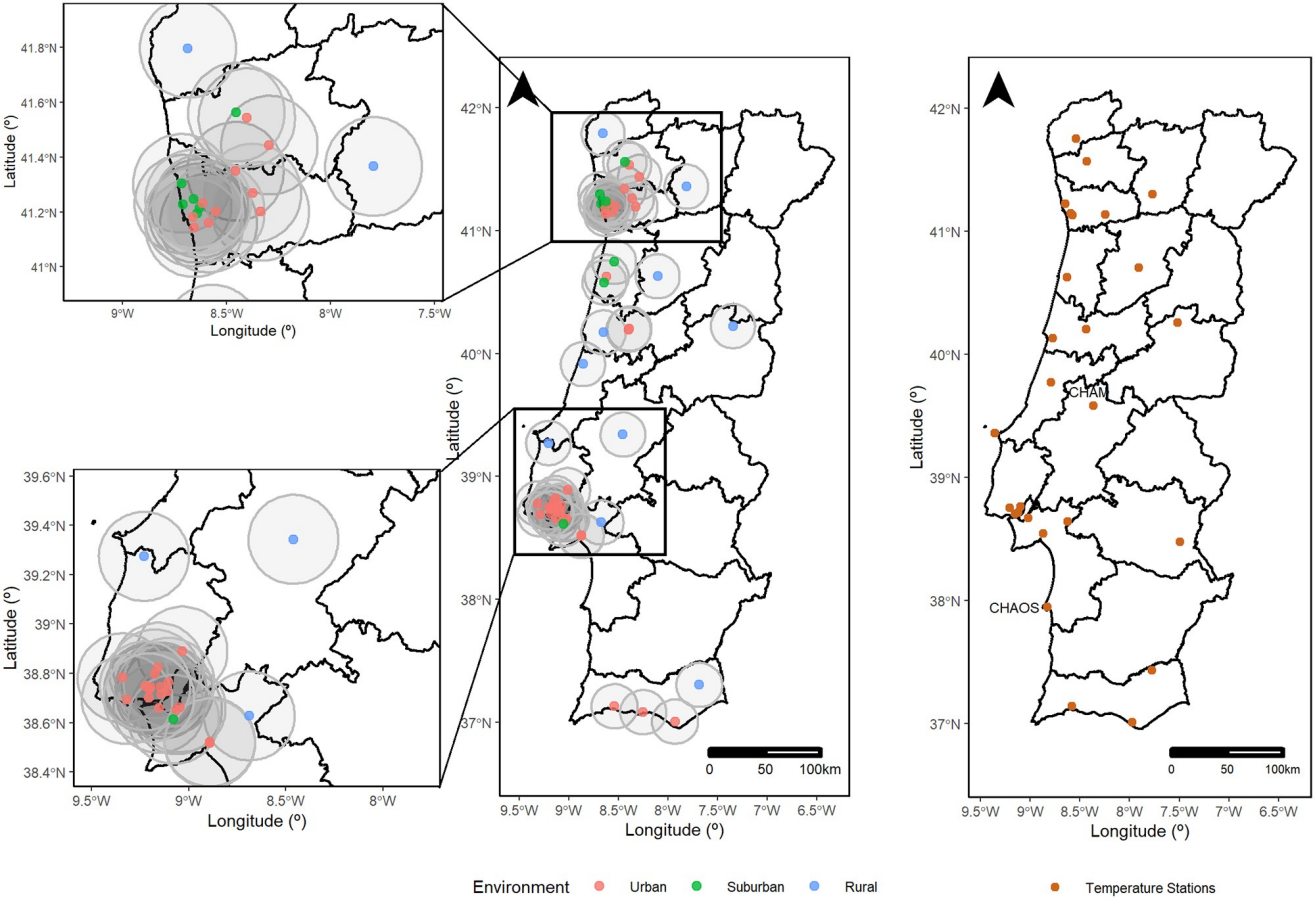
Portugal mainland maps and zooms over Lisbon and Porto districts. **(a)** air quality stations and **(b)** temperature monitoring stations. The grey circles represent the 20km radius of influence centered at each air quality station. The Portuguese limits and borders were constructed using the geographical information from the Carta Administrativa Oficial de Portugal 2017 (available for non commercial use at https://www.dgterritorio.gov.pt/cartografia/cartografia-tematica/caop).

Fig 3(a) displays the geographical location of the 58 air quality stations according to their type of environment: 35 urban, 10 suburban and 13 rural stations. Most of the urban stations are located either in Lisbon or Porto district which are zoomed in, respectively, in the lower and upper panels of Fig 3(a). In Lisbon, the largest metropolis in Portugal, all stations are urban with the exception of one suburban. Outside Lisbon, there are just a few rural stations. Porto district is the second largest metropolis, and it also holds a large proportion of urban stations. The countryside of Portugal has much less air quality monitoring stations, as the criteria to build such stations is based on population density [29] and the population density is considerably lower in the countryside. As illustrated in Fig 3(a) and 3(b), air quality and temperature data are not collected at the same geographical locations and the corresponding time series had to be paired based on their geographical proximity (euclidean distance between locations). The geographical distance between each one of the 58 air quality and the closest temperature station was, on average, of 8.5km, with a standard deviation of 6.9km. All distances vary between 40 meters (Monte Chãos, CHAOS) and 27.7km (Chamusca, CHAM) with 75% of the air quality stations exhibiting a distance to the paired temperature station lower than 14.3km.

### Statistical analysis

A detailed description of the methods used to perform the statistical analysis is presented. To this end, an overview of INGARCH models, their progressive development and the INGARCH model used in this work is initially presented. Then, the rational of the block-forward method for covariate selection is explained in detail. The code to use such method is available on GitHub as well as part on the (S1 File). Furthermore, we also make available a file with examples on how to use the block-forward method (S2 File). The usual tools for model assessment are introduced afterwards. Finally, one-step-ahead forecasts probability density function clustering is explained. The statistical analysis was conducted using R software version 3.6.2. Package tscount was used for modelling purposes and, cluster analysis was carried out using the HistDAWass package.

#### INGARCH models

A common approach to handle time series of counts is to consider INteger Generalized AutoRegressive Conditional Heteroscedastic (INGARCH) processes, where the serial dependence structure is incorporated through regression on past observations and past conditional means. The INGARCH process (*Y*_*t*_) of orders p,q∈N was firstly introduced by Heinen (2003) [19] and Ferland *et* al. (2006) [20] in which

$$\begin{array}{c} Y_{t}|F_{t-1}\;:\;\text{Poisson}\left(\lambda_{t}\right),\;\text{where}\;\lambda_{t}≔E\left(Y_{t}|F_{t-1}\right)=\beta_{0}+\sum_{k=1}^{p}\beta_{k}\;Y_{t-k}+\sum_{\ell =1}^{q}\alpha_{\ell }\;\lambda_{t-\ell }, \end{array}$$ (1)

being *Y*_*t*_, *t* ∈ {1, …, *n*} the count time series, $F_{t-1}≔\sigma \left(Y_{s},s\leq t-1\right)$ the history of the count process up to time *t* − 1. In addition, *β*_0_ > 0, *β*_*k*_ ≥ 0, *α*_*ℓ*_ ≥ 0, ∀_*k*,*ℓ*_ and $\sum_{k=1}^{p}\beta_{k}+\sum_{\ell =1}^{q}\alpha_{\ell }<1$, the latter condition to ensure that the INGARCH process is strictly-stationary. Tjøstheim (2012) further extended the above-model to incorporate link/transformation functions [21],

$$\begin{array}{c} g\left(\lambda_{t}\right)=\beta_{0}+\sum_{k=1}^{p}\beta_{k}\;\tilde{g}\left(Y_{t-k}\right)+\sum_{\ell =1}^{q}\alpha_{\ell }\;g\left(\lambda_{t-\ell }\right), \end{array}$$ (2)

where $g:R^{+}\to R$ is a link function and $\tilde{g}:N_{0}\to R$ is a transformation function. Such formulation of the model allows to deal with negative serial correlation. Also, Fokianos (2011) further considers the inclusion of a time-dependent covariate (*X*_*t*_) [23],

$$\begin{array}{c} g\left(\lambda_{t}\right)=\beta_{0}+\sum_{k=1}^{p}\beta_{k}\;\tilde{g}\left(Y_{t-k}\right)+\sum_{\ell =1}^{q}\alpha_{\ell }\;g\left(\lambda_{t-\ell }\right)+c\;X_{t}, \end{array}$$ (3)

where *c* is a real valued parameter. A recent work by Liboschik *et* al. (2017) expands this latter formulation to a matrix of time-dependent covariates ***X***_*t*_, *t* ∈ {1, …, *n*} [24],

$$\begin{array}{c} g\left(\lambda_{t}\right)=\beta_{0}+\sum_{k=1}^{p}\beta_{k}\;\tilde{g}\left(Y_{t-k}\right)+\sum_{\ell =1}^{q}\alpha_{\ell }\;g\left(\lambda_{t-\ell }\right)+\eta^{T}\;X_{t}, \end{array}$$ (4)

where ***X***_*t*_ = (*X*_*t*,1_, …, *X*_*t*,*r*_)^*T*^ is a time-varying *r*-dimensional covariate vector for each time *t* and ***η*** ≔ (*η*_1_, …, *η*_*r*_)^*T*^ is parameter vector of the covariates effects. In this last formulation, $F_{t-1}≔\sigma \left(Y_{s},X_{s+1},s\leq t-1\right)$ is the joint history of the process and of the covariates up to and including time *t*.

The recent implementation in R software [33] of the above-mentioned models allows the use of the Poisson or Negative Binomial distribution along with the identity or the logarithmic function [24]. The use of model (4) along with the Poisson distribution and the identity link results in an INGARCH model, whilst the use of the logarithmic function results in its log-linear extension. Due to its flexibility, in this work we restrict our attention to the Negative Binomial distribution and the logarithmic link/transformation functions in order to easily accommodate covariates into the model [24]. Therefore, the following representation of log(λ_*t*_) is used in the current analysis

$$\begin{array}{c} \text{log}\left(\lambda_{t}\right)=\beta_{0}+\sum_{k=1}^{p}\beta_{k}\;\text{log}\left(Y_{t-k}+1\right)+\sum_{\ell =1}^{q}\alpha_{\ell }\;\text{log}\left(\lambda_{t-\ell }\right)+\eta^{T}\;X_{t}, \end{array}$$ (5)

to avoid zero values [24]. In this model, $Y_{t}|F_{t-1}∼\text{NegBin}\left(\lambda_{t},\phi \right)$ with *ϕ* ∈ (0, ∞) representing the dispersion parameter. Note that $E\left(Y_{t}|F_{t-1}\right)=\lambda_{t}$ but the conditional variance is $Var\left(Y_{t}|F_{t-1}\right)=\lambda_{t}+\lambda_{t}^{2}/\phi$, where the limiting case *ϕ* → ∞ corresponds to the Poisson distribution. In this work, the matrix of time dependent covariates is defined with structure $X_{t}={\left(X_{t-k_{1},1},\ldots ,X_{t-k_{r},r}\right)}^{T}$, where each covariate can be considered in the model at a lag *k* that is not necessarily zero.

The model coefficients in (5) are estimated in a two step procedure as implemented in *tsglm* function of the *tscount* R package [24]. First, the *p* + 1 + *q* + *r* model parameters ***β*** ≔ (*β*_0_, …, *β*_*p*_)^*T*^, ***α*** ≔ (*α*_1_, …, *α*_*q*_)^*T*^ and ***η*** are estimated by maximising the conditional quasi log-likelihood function [24]. Second, given ${\hat{\lambda }}_{t}$ (i.e. the fitted values for λ_*t*_ obtained from $\hat{\beta }$, $\hat{\alpha }$ and $\hat{\eta }$), the dispersion parameter *ϕ* is then estimated by solving the equation based on the Pearson’s $X^{2}$–square statistic

$$\begin{array}{c} \sum_{t=1}^{n}\frac{{\left(Y_{t}-{\hat{\lambda }}_{t}\right)}^{2}}{{\hat{\lambda }}_{t}+{\hat{\lambda }}_{t}^{2}/\hat{\phi }}=n-\left(p+1+q+r\right), \end{array}$$ (6)

where *n* is the sample size. Note that this estimation procedure requires a fixed model order *p* and *q*. Optimal (*p*, *q*) pairs were automatically chosen by minimising the Akaike information criteria (AIC) for each of the 58 locations under analysis. Orders vary between 0 and 7 to accommodate several INGARCH-like structures and include terms related with the presence of weekly seasonality. With respect to the matrix of covariates, ***X***_*t*_ is iteratively constructed following the block-forward method detailed below. Briefly, the BF procedure automatically chooses each relevant covariate from the initial set of available covariates at the that lag maximises model performance.

The model in Eq (5) includes the information on both the history of the process (INGARCH part) and the covariates, and will be referred to as full model (M_F_, in short) throughout the text. Furthermore, two subfamilies of the M_F_ model are also considered: the pure INGARCH model (M_I_) by setting $\eta =\vec{0}$ and the reduced model (M_R_) by setting *p* = *q* = 0. Note that the M_I_ model ignores the covariates while the M_R_ model ignores the INGARCH component.

#### Block-forward method for covariate selection

This paper introduces a method for automatic selection of the time-varying covariates in matrix ***X***_*t*_ of the M_F_ model (5). The method is based on applying the forward approach to blocks of covariates, where at most one covariate from each block enters the full model. The method demands the *a priori* definition of the list of covariates, say *L*, that determines the composition and the order of the *b* sets of collinear covariates (blocks): *L*[[*i*]][*j*] stores one covariate at the position *j* of the block *i* for *i* = 1, 2, …, *b* and *j* = 1, 2, …, *N*_*i*_ where *N*_*i*_ is the number of covariates in the *i*^*th*^ block. The covariates included in one block are expected to be correlated at a large extent, thus showing a similar (empirical) effect on *Y*_*t*_ counts. The order of the blocks should reflect the (empirical) relevance of the covariates in explaining the target count process *Y*_*t*_, thus, the covariates in the first blocks are expected to be the most associated with the outcome.

It is known that the delayed/lag effect of a given air pollutant in daily hospital admissions depends, e.g., on the pollutant itself and on the geographical location, showing lag values typically lower than 7 days [34]. Therefore, the effect of a given covariate on *Y*_*t*_ was considered at different time lags, by allowing the structure ***X***_*t*_ = (*X*_*t*−*k*_1_, 1_, …, *X*_*t*−*k*_*r*_, *r*_)^*T*^ for the matrix of covariates in model (4) with *k*_*l*_ ≤ 7, *l* = 1, 2, …, *r*. Thus, while defining the list *L*, a covariate and also their lagged versions (up to lag 7) were considered in the same block. In a given block, either a covariate or one of their lagged versions is allowed to enter the model.

**Fig 4:**
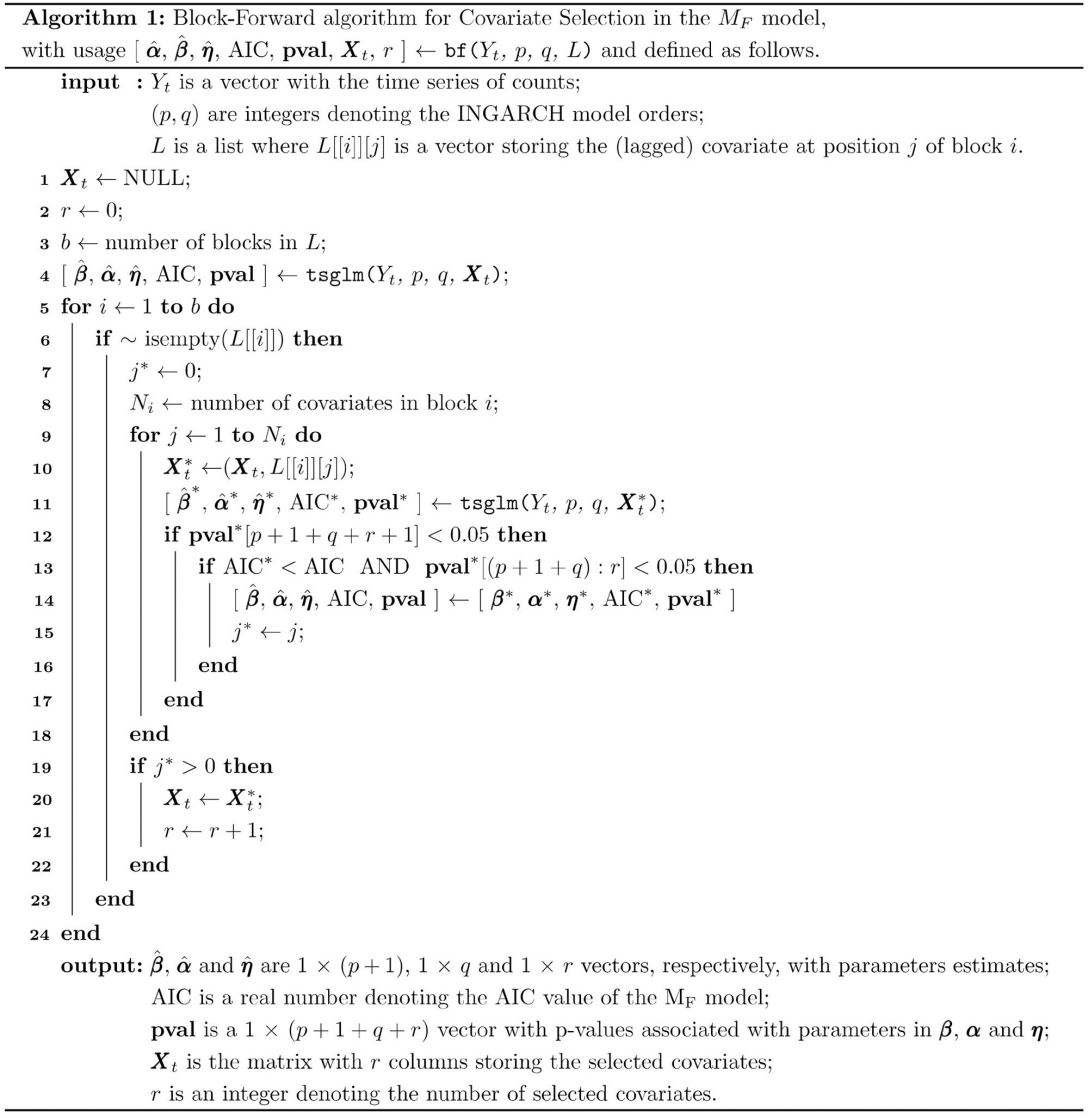
Algorithm outline for the block-forward approach.

The algorithm is outlined in Fig 4. The algorithm starts by fitting the M_I_ model with optimal (*p*, *q*) orders to (*Y*_*t*_). At this stage, M_I_ model does not include any covariates (or lagged versions) and will serve as the initial current model (null model) to decide the inclusion of a covariate of the first block in the M_F_ model. The following procedure is then performed to each block *i*. For each covariate delayed at a given lag, the effect of adding such covariate into the current model is quantified from the AIC of the candidate model (i.e. the current model with the add-on covariate) and the *p*-value associated to the covariate coefficient. The significant covariate (*p*-value <0.05) of the block leading to the candidate model with lowest AIC value among all candidate models is selected to enter the current model, as long as the coefficients of the covariates from the previous blocks included in the model remain statistically significant. The M_F_ model is then updated by adding the selected covariate to the current model of the algorithm. The algorithm stops when all blocks are inspected. At the end, the updated ***X***_*t*_ matrix will include the selected *r* ≤ *b* significant covariates.

**Fig 5:**
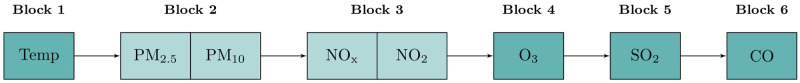
Blocks of covariates in the block-forward approach.

The blocks in Fig 5 were defined to assess the effect of temperature and air pollutants on respiratory hospital admissions. This structure corresponds to the definition of the list *L* following *L*[[1]][1] = TEMP(*t*), *L*[[1]][2] = TEMP(*t* − 1), …, *L*[[1]][8] = TEMP(*t* − 7), *L*[[2]][1] = PM_2.5_(*t*), …, *L*[[2]][9] = PM_10_(*t*), …, *L*[[2]][16] = PM_10_(*t* − 7), *L*[[3]][1] = NO_x_(*t*) and so on. Moreover, each block is composed by the covariates that are expected to be associated and to induce the same (empirical) effect on hospital admissions. Furthermore, the order of the blocks reflects the current knowledge on the magnitude of the effect of temperature and air pollutants on hospital admissions.

The first block solely includes temperature which is known to have an important effect on mortality, although its impact on hospital admissions is much less understood [35]. Studies on the association of both low and high temperatures, and respiratory hospital admissions have found a positive association [35–38]. The Assessment and Prevention of Acute Health Effects of Weather Conditions in Europe (PHEWE) project, which studied both the short-term effect of cold and hot weather on respiratory hospital admissions in 12 European cities, reported a significant effect of minimum temperatures observed for all ages in the North-Continental cities (-2.5%, 95%CI: -3.6, -1.3) and Mediterranean cities (-1.6%, 95%CI: -2.5, -0.6) [37]. The project also report a positive association between respiratory admissions and the maximum temperature: for an increase of 1°C degree in maximum apparent temperature above a selected threshold, respiratory admissions increased by +4.5% (95%CI: 1.9–7.3). When the overall effect of mean temperature on hospital admissions is analysed (i.e., annual effect) a negative association is reported [26].

The second block includes the particulate matter covariates: PM_2.5_ and PM_10_. These air pollutants are linked to worse health outcomes due to their ability to get into the thoracic region and by getting deposited in the smaller conducting airways and alveoli [27]. These air pollutants are correlated since, by definition, PM_10_ can also include PM_2.5_. A recent systematic review showed that PM (either PM_2.5_ or PM_10_) have higher influence on respiratory hospital admission than the other air pollutants [39], which supports their place after temperature and before the remaining air pollutants. Similarly to the PM, NO covariates were included in one block. These covariates are expected to be correlated since NO_2_ is one of the main constituents of NO_x_. Both nitrogen oxides are originated by the combustion processes in stationary sources (heating, power generation) and in mobile sources (internal combustion engines in vehicles and ships) and these might have effects on lung metabolism, structure, function, inflammation and host defence against pulmonary infections [27]. Also, these pollutants had the second highest correlation with hospital admissions [39]. Finally, the air pollutants O_3_, SO_2_ and CO were considered in individuals blocks as their effect on hospital admission may be independent, in the sense that they capture distinct information, unlike PM or NO covariates. O_3_, contrarily to the remaining air pollutants, is not directly emitted from primary sources. There is evidence that this air pollutant affects inflammatory pathways, but the evidence of association between O_3_ and respiratory hospital admissions is not consistent, although several studies suggest a positive association [27]. Sulphur dioxide (SO_2_) results mainly from combustion. There is considerable evidence suggesting acid aerosols derived from sulphur dioxide emissions, contributes to exacerbation of asthma by worsening its symptoms and reducing lung function. There are studies that show an association between SO_2_ and respiratory hospital admissions, but there is uncertainty as to whether sulphur dioxide may work as surrogate for ultrafine particles, since they have common sources [27]. Hence, positioning SO_2_ after PM allows to mitigate this uncertainty. Finally, CO is considered in the last block, since a recent systematic review showed that this pollutant was not associated with respiratory hospital admissions [39]. Nevertheless, as this pollutant has known toxicological characteristics on human health it was considered in the analysis [27]. Note that whilst most studies consider the mean of air pollutants and temperature, we consider the maximum daily value, which may allow to better identify the effect of these covariates on respiratory hospital admissions.

The M_F_ model constructed for each spatial location is conditioned by the availability of a given covariate at that location. For instance, PM_2.5_, SO_2_ and CO were not monitored at Valongo location during the time period under analysis (see S4 Table). In the Valongo case, *L*[[1]][2], *L*[[5]][1] and *L*[[6]][1] in Fig 4 are empty entries and, thus PM_2.5_, SO_2_ and CO could not be considered to enter the M_F_ model for that location.

#### Model assessment

Models’ adequacy was investigated through Pearson’s residuals defined as

$$\begin{array}{c} r_{t}=\frac{Y_{t}-{\hat{\lambda }}_{t}}{\sqrt{{\hat{\lambda }}_{t}+{\hat{\lambda }}_{t}^{2}/\hat{\phi }}}, \end{array}$$ (7)

where ${\hat{\lambda }}_{t}$ and $\hat{\phi }$ correspond to the ML estimates of λ_*t*_ and *ϕ*, respectively. If the fitted model is correctly specified, Pearson’s residuals should be uncorrelated and normally distributed with zero-mean and unit-variance. Another useful tool to evaluate models’ adequacy, namely the accordance between a probabilistic forecast and the observation, is the probability integral transform (PIT) [40]. If the predictive distribution is correct, then the PIT representation should follow a standard uniform distribution. For count data, a non-randomised PIT value for *Y*_*t*_ with predictive distribution *P*_*t*_(*Y*) is defined as

$$\begin{array}{c} F_{t}\left(u|Y\right)=\{\begin{array}{cc} 0 & \text{if}\;u\leq P_{t}\left(Y\right)\\ \frac{u-P_{t}\left(Y-1\right)}{P_{t}\left(Y\right)-P_{t}\left(Y-1\right)} & \text{if}\;P_{t}\left(Y\right)-P_{t}\left(Y-1\right)<u<P_{t}\left(Y\right),\\ 1 & \text{if}\;u\geq P_{t}\left(Y\right) \end{array} \end{array}$$ (8)

where $u≔P_{t}\left(Y-1\right)+v\left[P_{t}\left(Y\right)-P_{t}\left(Y-1\right)\right]$ and *v* is the standard uniform. The mean PIT is estimated by $\bar{F}\left(u\right)=\frac{1}{n}\sum_{t=1}^{n}F_{t}\left(u|Y_{t}\right)$, 0 ≤ *u* ≤ 1 and converted into the empirical PIT histogram which simplifies the comparison with the standard uniform (i.e., a flat line) [40]. The histogram is computed with *J* = 10 equally spaced bins with heights $f_{j}=\bar{F}\left(j/J\right)-\bar{F}\left(\left(j-1\right)/J\right)$ for *j* = 1, …, *J*. A U-shape indicates underdispersion of the predictive distribution, while an upside down U-shape indicates overdispersion [24].

#### Forecasting and clustering

The probability density function (pdf) of the one-step-ahead forecast for *Y*_*n*+1_ was estimated for each location. To this end, 10 000 sample paths of the M_F_ model fitted to each location were generated. For each path, the optimal forecast ${\hat{Y}}_{n+1}$, in terms of the mean squared error, was obtained by computing the conditional mean ${\hat{\lambda }}_{n+1}$. The evaluation of ${\hat{\lambda }}_{n+1}$ is straightforward from Eq (5) by plugging-in the history of the path, the values of the covariates and the ML estimates for the *β*_1_, …, *β*_*k*_ and *α*_1_, …, *α*_*ℓ*_ model coefficients and also the estimate of *ϕ*. The one-step-ahead forecasts obtained for all paths were then used to estimate the pdf of the one-step-ahead forecast of that location. Next, the pairwise distance between the pdfs estimated for the 58 locations is calculated through *ℓ*^2^-Wasserstein distance [41]. The results of the clustering procedure are illustrated through a dendrogram based on Ward’s agglomerative method for the grouping criterion, which corresponds to a sum-of-squares criterion that estimates groups that minimise the within-group dispersion at each binary fusion [42]. The optimal number of clusters was evaluated from the Mojena’s upper tail statistics [43], defined as

$$\begin{array}{c} a_{j}^{*}=\frac{a_{j}-{\overset{-}{a}}_{j-1}}{s_{j-1}}, \end{array}$$ (9)

where *a*_*j*_ represents the linkage distance at level/class *j* and ${\overset{-}{a}}_{j-1}$ and $s_{{\overset{-}{a}}_{j-1}}$ are the average and standard deviation of the linkage distances at the *j* − 1 previous fusion levels. The existence of an elbow on the (*j*, *a*_*j*_) plot at the value *j* = *k* suggests that *k* is the adequate number of clusters to consider, whereas the absence of an elbow suggests that there is no relevant classification [44].

## Results and discussion

**Fig 6:**
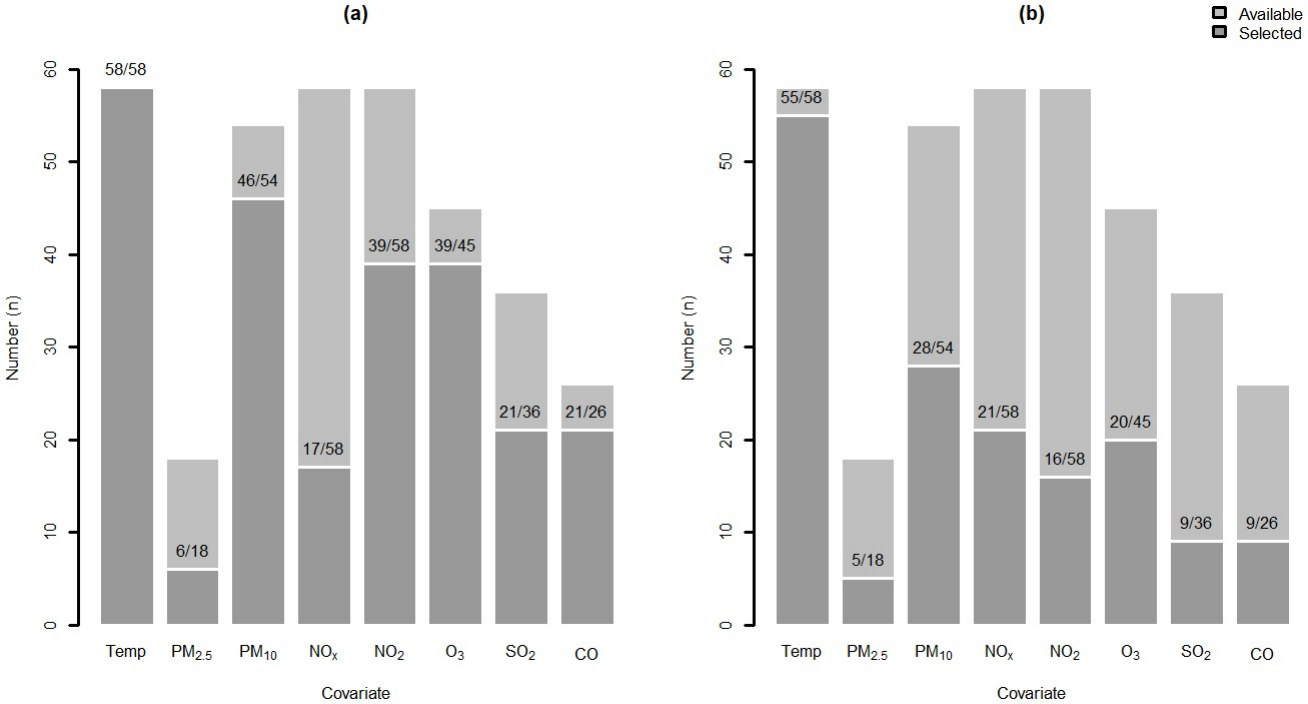
Barplot with the number of models with significant covariates (dark grey) out of the number of models with available covariates (light grey): **(a)** Reduced model M_R_ and **(b)** Full model M_F_. The total number of spatial locations is 58.

A reduced M_R_ and a full model M_F_ were fitted to the data referring each of the 58 spatial locations. Fig 6 shows the number of M_R_ and of M_F_ models for which a given covariate was available and selected. Temperature, NO_x_ and NO_2_ are available at all locations. Concerning the PM covariates, the 18 locations with PM_2.5_ available also have PM_10_ data and, overall, PM information is accessible at 93% (54/58) of the locations. The corresponding percentages for O_3_, SO_2_ and CO are respectively 78% (45/58), 62% (36/58) and 45% (26/58), thus being the covariates less available for model selection. From the comparison between the dark grey bars in Fig 6(a) and 6(b) it stands out that covariates are more often selected in M_R_ than in M_F_ models; this was expected as M_R_ neglects the past information of the time series of hospital admissions. Temperature is selected in all M_R_ models and in 55/58 (95%) of the M_F_ models. With respect to PM, either PM_2.5_ or PM_10_ is selected in 96% (52/54) of M_R_ and in 61% (33/54) of M_F_ models. Turning now to the NO covariates, either NO_x_ or NO_2_ is selected in 97% (56/58) and in 64% (37/58) of the M_R_ and the M_F_ models, respectively. The results for M_F_ models show no evident preference between NO_x_ or NO_2_, in accordance with the fact that both covariates hold similar information and that are expected to be highly correlated (e.g. correlation between NO_x_ and NO_2_ achieving 0.96 with data collected in Poland [45]). The remaining pollutants O_3_, SO_2_ and CO are selected in 87% (39/45), 58% (21/36) and 81% (21/26) of the M_R_ models, respectively. The corresponding M_F_ values are 44% (20/45), 25% (9/36) and 34% (9/26). Despite being less available and less times selected, the effect of O_3_, SO_2_ and CO still remain significant to explain the time series of hospital admissions beyond the effect of temperature, PM and NO. In particular for Valongo (VALO) location, both M_R_ and M_F_ include temperature as well as PM, NO and O_3_ covariates. This result clearly establishes that air quality is associated with hospital admissions due to respiratory causes, even when the past history of the count process is incorporated into the statistical model.

The number of available and selected covariates was also evaluated by taking into account the type of environment (urban, suburban and rural) and the type of influence (background, industrial and traffic) in each location. Table 1 shows that the number of covariates per block from the M_R_ to the M_F_ model decreases, regardless of the type of environment or influence. With respect to the M_F_ model, temperature is equally selected in monitoring stations with different type of environments and influence, which supports that temperature is an important covariate to model daily hospital admissions due to respiratory causes, beyond the effect of the history of the process. On the contrary, CO is a covariate available and not selected to characterise the daily hospital admissions due to respiratory causes at rural and, its expression is residual at suburban locations (1/10). The results according to the type of influence, neither suggest the predominance nor absence of a given air pollutant. However, PM_2.5_ is selected in locations where it is predominately available, i.e. background influence stations (S4 Table). Nevertheless, it is important to stress that the majority of M_F_ models (55/58) select, at least, one air quality covariate (S4 Table). This result reinforces that the effect of air pollutants is significant and relevant to explain respiratory related daily hospital admissions, despite the type of environment or influence considered. See S4 Table for the indication of available/selected covariates for all M_F_ models.

**Table 1:** Number of selected covariates in the M_R_ and M_F_ models stratified by type of environment and type of influence.

		M_R_								M_F_							
	Total	Temp	PM_2.5_	PM_10_	NO_x_	NO_2_	O_3_	SO_2_	CO	Temp	PM_2.5_	PM_10_	NO_x_	NO_2_	O_3_	SO_2_	CO
**Environment**																	
Urban	35	35	0	31	8	27	21	10	17	33	1	17	12	10	9	5	8
Suburban	10	10	0	9	4	6	8	5	4	10	1	6	4	2	6	0	1
Rural	13	13	6	6	5	6	10	6	0	12	3	5	5	4	5	4	0
**Influence**																	
Background	37	37	6	28	12	23	30	13	7	34	5	18	11	10	15	7	2
Industrial	7	7	0	5	2	5	6	4	4	7	0	3	2	2	4	1	2
Traffic	14	14	0	13	3	11	3	4	10	14	0	7	8	4	1	1	5
Total Selected		58	6	46	17	39	39	21	21	55	5	28	21	16	20	9	9
Total Available		58	18	54	58	58	45	36	26	58	18	54	58	58	45	36	26

**Fig 7:**
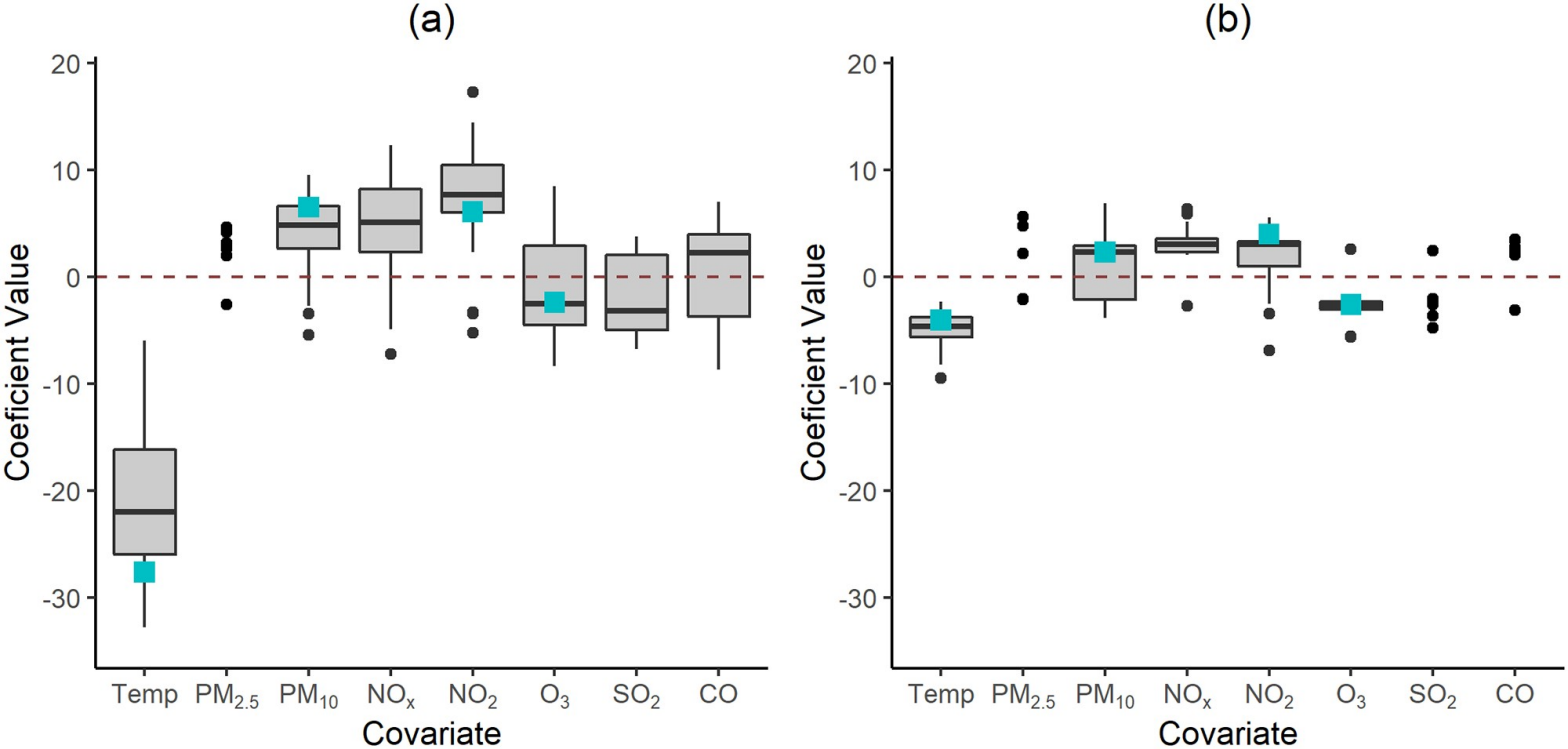
Distribution of the scaled coefficients according to each covariate. **(a)** M_R_ and **(b)** M_F_ models. Boxplots are presented when there are at least 15 locations otherwise each dot represents a location. The blue squares identify the coefficients estimated for Valongo (VALO) location.

This study also explored the effect size of each covariate in daily hospital admissions. The available literature has long demonstrated the effect of outdoor temperature on the outcome where higher risk is associated with more extreme temperatures (see e.g. [32]). This is not the case of the effect of air pollutants which have not been as extensively studied. Fig 7 displays the distribution of the scaled coefficients estimated for each selected covariate (i.e., the coefficient divided by its standard error), which are unit-free and, thus comparable in terms of magnitude among all covariates. The effect of temperature as assessed from M_R_ models is much larger than that of the remaining covariates, which is consistent with the literature [39]. However, when the history of the count process is fed to the model, the effect of temperature assessed from the M_F_ models is closer to that of air pollutants, suggesting that, a considerable proportion of its effect is retained in the history of the count process. In both models, the temperature coefficient is negative indicating that the lower the temperature, the higher the logarithm of the hospital admissions is. These models provide the (annual) average effect of a covariate; hence, in Portugal, the effect of lower temperatures on respiratory hospital admissions is predominant on winter in contrast to those of higher temperatures during the summer. On the contrary, PM and NO covariates have, in general, positive coefficients showing that an increase in the concentration of these pollutants leads to an increase in hospital admissions. Ozone is negatively correlated with hospital admissions i.e. lower levels of O_3_ are associated with increasing hospital admissions. One reason for such negative association may be the conversion reaction of O_3_ into NO_2_ [27]. With respect to SO_2_ and CO, although median associations with hospital admissions are respectively negative and positive for the M_R_ models, there is no clear association pattern (either negative or positive) for the M_F_ models, which might result from the type of influence of each monitoring station and the low concentrations registered over the last decades for both pollutants [46]. With regards to Valongo location (blue squares), the coefficients present the expected direction of association. In accordance with the overall results, the coefficients have higher absolute values for the M_R_ model than for the M_F_ model.

**Fig 8:**
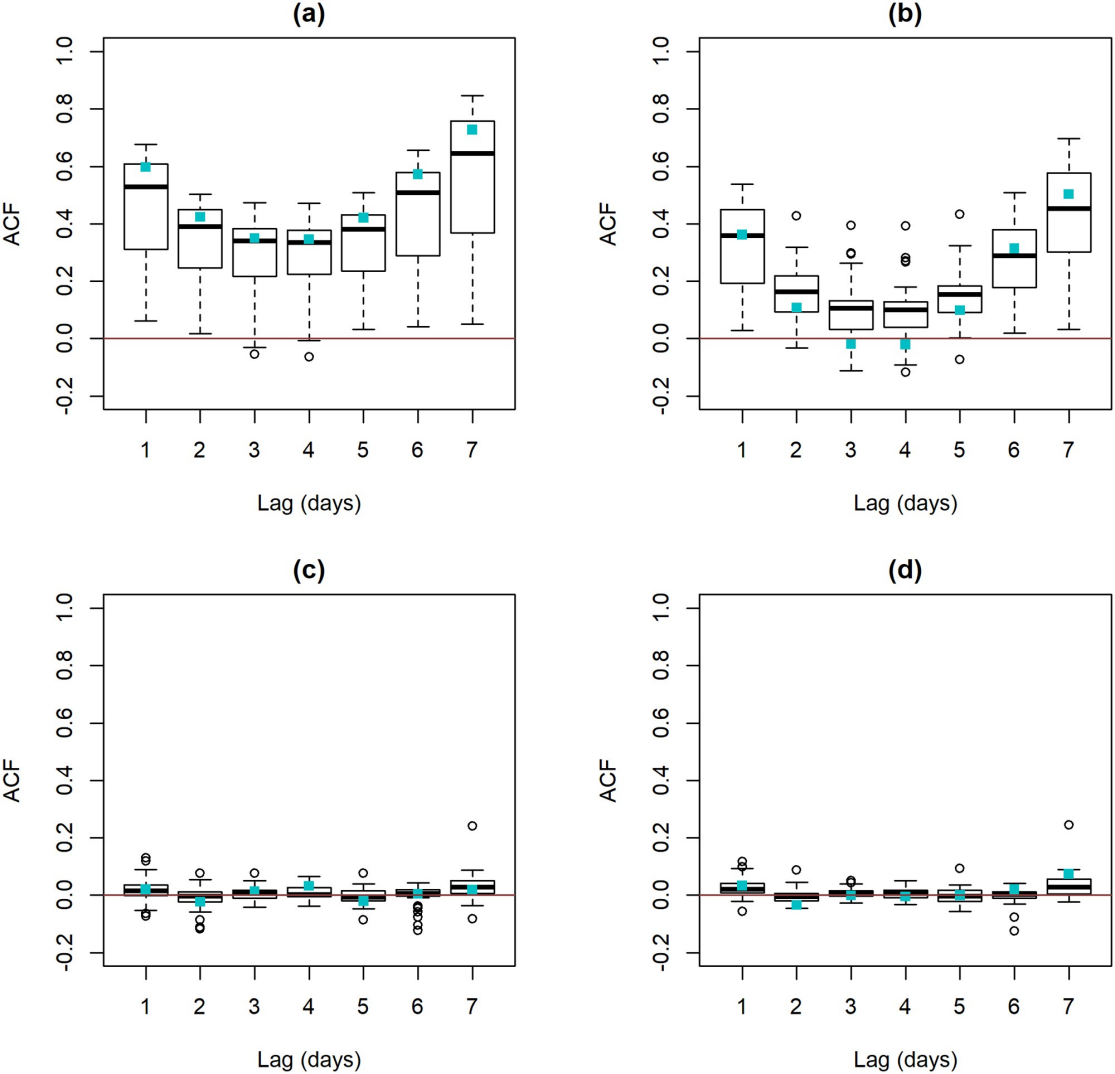
Boxplot of sample ACF values calculated at each location. **(a)** the time series of counts with daily hospital admissions, **(b)** residuals of the M_R_ models, **(c)** residuals of the pure INGARCH M_I_ models and **(d)** residuals of M_F_ models. The blue squares identify the sample ACF values for Valongo (VALO) location.

Fig 8 displays the distribution of sample ACF values across the 58 spatial locations. As presented in Fig 8(a), the time series of the number of daily hospital admissions exhibits considerable non-zero autocorrelation values. This representation also shows higher variability in the sample ACF values by location for lags 1, 2, 6 and 7 when compared with the remaining lags. The sole impact of the covariates in the daily admissions can be assessed by comparing Fig 8(a) and 8(b): the sample ACF values of the M_R_ residuals are smaller than those of the original time series of counts, thus suggesting that the covariates indeed explain part of the variability of hospital admissions. Nevertheless, much of the data variability remains to be explained. The impact of the history of the count process in the modelling is illustrated in Fig 8(c) and 8(d). On one hand, the sample ACF of the M_I_ residuals is remarkably lower than the sample ACF of the original data, thus showing that the history of the process itself explains a large part of the information. On the other hand, the sample ACF values of the M_I_ and M_F_ residuals are fairly similar, evidencing the small impact of the covariates in hospital admissions beyond that of the history of the process. Despite having a small impact when compared with the history of the count process it is worthwhile to note that the effect of the covariates is statistically significant. Finally, the sample ACF values for Valongo location (represented by the blue squares) are consistent with the overall results described. As illustrated in Fig 8(a) and 8(b), it is quite clear that the sample ACF values of the M_R_ residuals are substantially smaller than those of the original series, in particular between lags 2 and 6 days. Furthermore, the sample ACF values of the M_F_ residuals are quite close to zero, regardless of the lag.

The sample ACF of the residuals also allows the evaluation of the model adequacy to the data. Overall Fig 8(d), shows that the M_F_ residuals do not exhibit any relevant serial correlation or seasonality which has not been taken into account by the M_F_ models. This result also reinforces that both the history of the count process and the covariates are able to explain, at a large extent, the temporal patterns in daily hospital admissions. Finally, the mean and the variance of the M_F_ Pearson’s residuals were close to the target values zero and one, respectively, showing the sample average ± standard deviation values of -0.005 ± 0.007 and 0.994 ± 0.002 across the 58 spatial locations.

**Fig 9:**
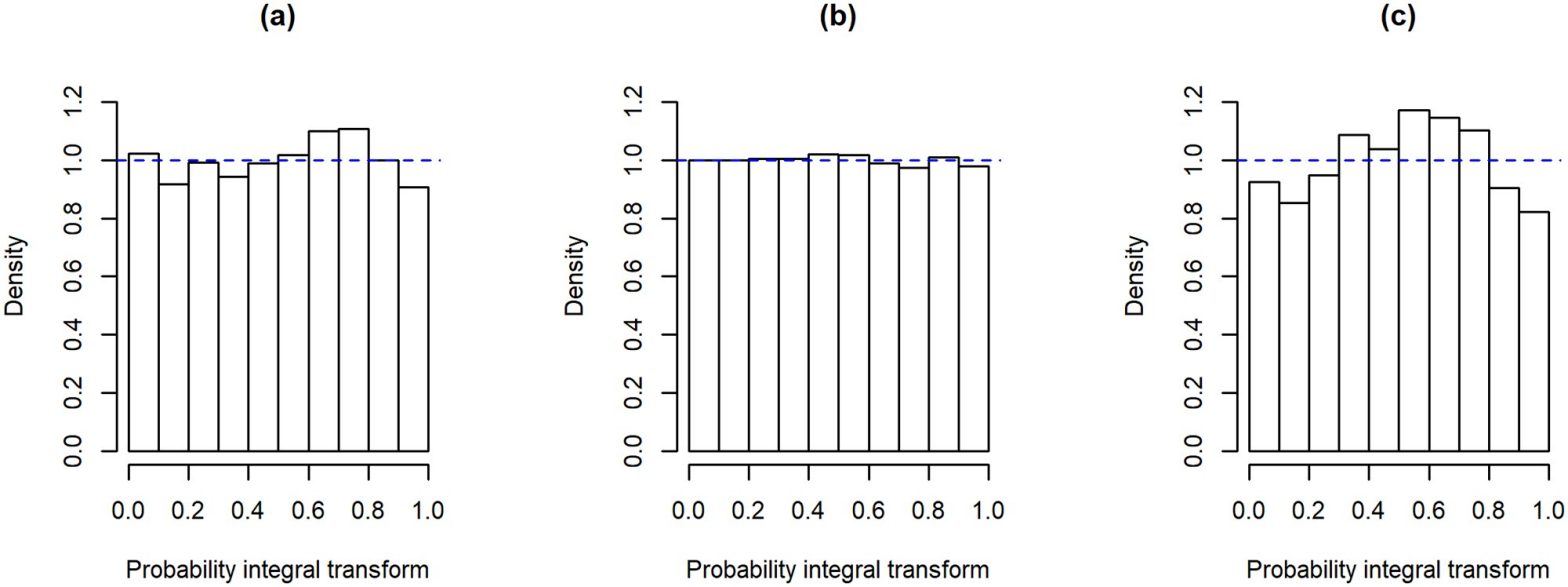
PIT histograms for the M_F_ model in 3 locations. **(a)** Valongo, **(b)** location with PIT closest to uniformity and **(c)** location with PIT furthest to uniformity.

The PIT histograms for the M_F_ models are further used to investigate the adequacy of the Negative Binomial distribution as being the predictive one. Fig 9(a) shows the PIT histogram for Valongo suggesting that the probabilistic calibration of the Negative Binomial model is satisfactory for this location. Fig 9(b) and 9(c) show the PIT histograms for the locations with PIT closest/furthest to uniformity. These figures provide an insight of the range of the deviations from the uniform distribution across locations.

**Fig 10:**
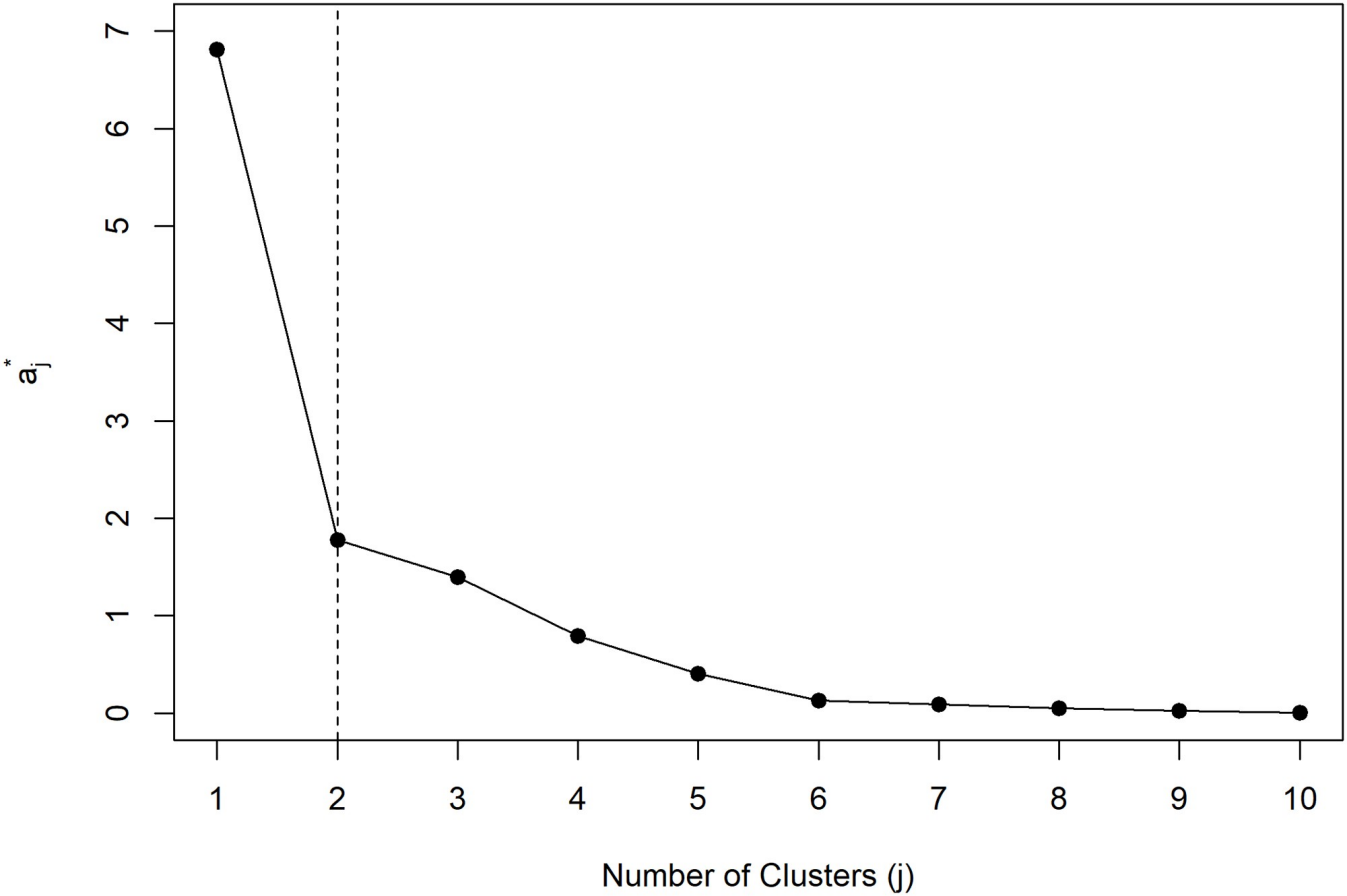
Plot of the Mojena’s statistics as a function of the number of clusters.

**Fig 11:**
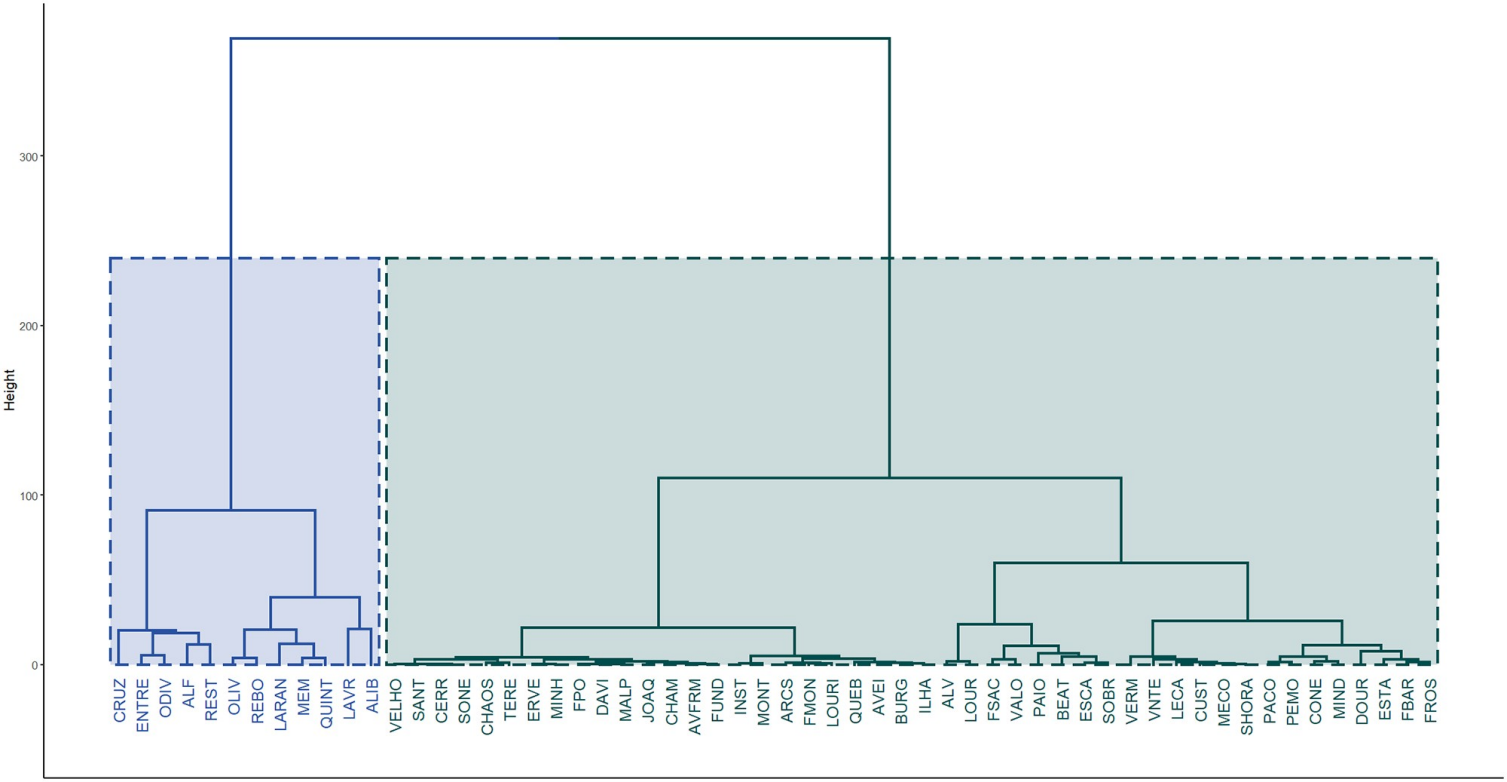
Dendrogram of the estimated one-step-ahead forecast pdfs highlighting the identified clusters. **(a)** Cluster 1 (blue) and **(b)** Cluster 2 (green).

After the validation of the M_F_ model, the pdf of the one-step-ahead forecast was computed for each location. The resulting cluster analysis of the 58 estimated pdfs considered 2 groups, as suggested by the location of the elbow for the Mojena’s rule values represented in Fig 10. Fig 11 shows the corresponding dendrogram highlighting the two clusters, that exhibit small within group dispersion (i.e. high similarity among pdfs in the same cluster) and large intra group differences (i.e. low similarity among different clusters). The cophenetic correlation coefficient of 0.90 indicates that the dendrogram’s represents rather well the matrix of pairwise distances between estimated pdfs at different locations.

**Fig 12:**
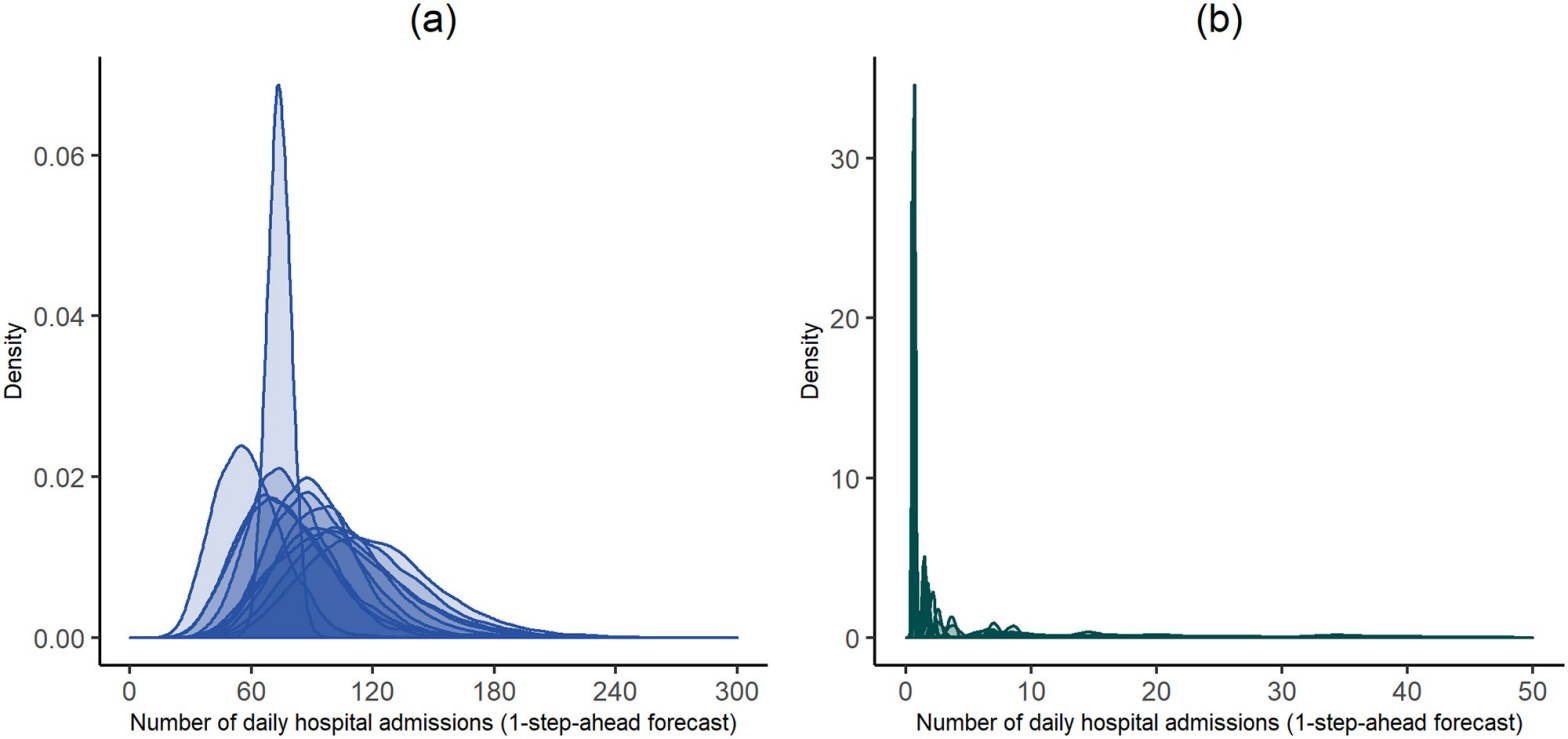
Estimated one-step-ahead forecast pdfs stratified by identified clusters. **(a)** Cluster 1 (blue) and **(b)** Cluster 2 (green). The scale of the *x*-axis decreases by a factor of 6 from one cluster to other.

Fig 12 shows the estimated one-step-ahead forecast pdfs per identified cluster. Cluster 1 (blue) includes, exclusively, locations from the Lisbon metropolitan area, exhibiting higher forecast values for the daily hospital admissions and larger variability than those from Cluster 2 (green). It is worthwhile to notice, however, that there is one location (ALIB) that stands out from all the pdfs in Cluster 1, exhibiting a quite smaller variability with respect to the pdfs of the same cluster. On the contrary, Cluster 2 is characterised by locations exhibiting low (on average <10) daily hospital admissions and moderate one-step-ahead forecasts (on average<40) daily hospital admissions with high probability. Within this cluster, these two subgroups can be identified: the left subgroup corresponds to the low forecasts and, the right subgroup corresponds to the moderate one-step-ahead forecast probabilities (Fig 12). The subgroup with moderate one-step-ahead forecast probabilities contains urban and suburban location, whereas the other group also contains rural locations. Therefore, the cluster analysis shows that locations in Cluster 1 are more likely to have a higher number of daily hospital admissions than those in Cluster 2. This is expected because the monitoring stations in Cluster 1 are all urban and located in Lisbon metropolitan area, and thus their surrounding areas exhibit much higher overall levels of air pollutants (S2 and S3 Tables) and much higher population density.

It is important to address some of the potential limitations of the present study. One of the critical choices in this analysis concerns the 20km radius of the influence circumference defined around each monitoring station, to produce the paired time series of the number of daily hospital admissions. We pursued a sensitivity analysis on the M_F_ models constructed based on time series of counts produced for smaller and larger radius (namely 10km, 15km and 25km). The models/results were found to be similar for the different radius not changing the conclusions of this analysis. However, it is likely that the model obtained for one given location could benefit from an location-adjusted radius, reflecting its real surrounding environment and influence (e.g. traffic stations are expected to be better represent narrow radius). Another important issue that deserves a remark is whether the influence areas with substantial overlapping result in similar models (see e.g. zoom over Lisbon district in Fig 3). To pursuit this issue it is important to realise that the availability of air pollutants changes from one influence area to another despite their proximity. Furthermore, the time span covered can also vary between influence areas, which could result in different available air pollutants at different temporal moments. Another factor influencing the set of covariates is the quality of the data acquisition, as some air pollutants were excluded from this analysis due to their large periods of missing data (most likely as a result of probes malfunctioning). As expected, M_F_ models were found to be similar when associated with relatively close (or overlapping) influence areas, covering the same time span and with the same available covariates. Examples of such models are those associated with Aveiro (AVEI) and with Ílhavo (ILHA) monitoring stations, 5km apart from each other, where both models include temperature, PM_10_ and a NO covariate (see S4 Table). ILHA additionally selects O_3_ and SO_2_ for the M_F_ model, which are not available at AVEI monitoring station, while AVEI selects CO, which is unavailable at ILHA. Therefore, it is important to put emphasis on the fact that the models are build with the available covariates at each geographical location which may lead to different model structures even for close/overlapping influence areas.

The impact of the Block-Forward procedure with 6 ordered blocks (see Fig 5) in building M_F_ models was assessed by comparing the AIC and the number of significant covariates among models produced by different BF-based strategies (6 ordered blocks, 8 ordered blocks and 6 non-ordered blocks). As shown in Table 2, the AIC values and the number of significant covariates were similar for all approaches, over the 58 spatial locations. The similar AIC values and number of covariates selected among the different BF based models clearly shows that the 6 ordered block strategy (imposing blocks of covariates and their order) does not lead to lower performance of the models (which might restrict the data analysis) and constitutes a more standardised framework to analyse the different spatial locations. The 6 ordered block models were also compared to saturated models, which exhibit similar AIC values but have a higher number of significant covariates. This result highlights the importance of using the BF with 6 ordered blocks as a covariate selection procedure in this applied setting, since the M_F_ models exhibit higher parsimony. As a final note, the results above support the inclusion of empirical knowledge within the BF approach. The BF procedure allows for a more systematic model construction by restricting the blocks’ order. Furthermore, the interpretation of PM and NO covariates is clearer since they are considered in blocks, and only one enters the model. Hence, the inclusion of empirical knowledge within the block-forward approach leads to a valid, comprehensible and systematic procedure for covariate selection.

**Table 2:** AIC and number of significant covariates (#) for the BF based and the saturated models over the 58 locations. The AIC is displayed with distributional quartiles Q1, Q2 (median) and Q3, and # is shown as mean ± standard deviation.

Approach	AIC Q2 (Q1, Q3)	# mean ± sd
**M_F_ models**		
BF 6 ordered blocks	16864 (13843, 19163)	2.81 ± 1.02
**Other BF models**		
8 ordered blocks	16863 (13834, 19163)	3.09 ± 1.27
6 non-ordered blocks	16861 (13839, 19144)	2.74 ± 1.19
**Saturated models**	16852 (13863, 19153)	2.98 ± 1.08

M_F_ models are constructed from BF with 6 ordered blocks (see Fig 5). BF with 8 ordered blocks results from splitting blocks 2 and 3 into 4 blocks. In the BF with 6 non-ordered blocks, the significant covariate entering the model, at each step, is that with minimum AIC on the current model. Saturated models include all covariates (regardless if significant or not).

## Conclusion

The overall goal of this work was to conduct a comprehensive study on the effect of air pollution, beyond the effect of temperature, on respiratory hospital admissions in Portugal mainland. We found that models including only covariates are able to describe some variability on daily respiratory hospital admissions. However, when models comprise the history of the hospital admissions, they are able to explain a considerably larger amount of variability since information on the dependence structure of the count time series itself is now included. From all the covariates considered, temperature, as expected, is the most determinant covariate. Nevertheless, after considering the past information of the process, air quality still adds important information to the model. Hence, we conclude that there exists a significant association between hospital admissions and air quality beyond the effect of count time series history and temperature. None of the environmental covariates was found to be predominant in all INGARCH models, even when analysing by type of environment and influence of the corresponding monitoring station, suggesting that general actions to improve air quality are needed across the country. Furthermore, the cluster analysis showed that higher counts of daily hospital admissions are more likely in the urban locations of the Lisbon metropolitan area. This result highlights that special attention must be given to air quality in Lisbon metropolitan area in order to achieve a relevant decrease in the number of hospital admissions. Finally, this work also contributes to covariate selection strategies by successfully implementing the block-forward strategy which can be used in multiple settings and has the following advantages: account for collinearity, deal with missing covariates and consider current empirical knowledge.

Summing up, this work adds to the current body of knowledge of the effect of air quality on respiratory hospital admissions by using INGARCH-type models. These models are not broadly used in this setting although they can adequately model the outcome variable (respiratory hospital admissions). Hence, these results advocate for the use of a time series model approach when analysing the effect of air quality on health in contrast with other approaches, such as the commonly used GAM models.

## Supporting information

S1 File R code for block-forward method. This file provides the code for using the block-forward method within the *tscount* package in R version 3.6.2.
(R) Click here for additional data file.

S2 File R code with an application example of the block-forward method. This file provides examples on how to use the block-forward method.
(R) Click here for additional data file.

S1 Table Indication of the time period, location and type of environment (urban, suburban and rural) and of influence (background, industrial and traffic). (PDF)

S2 Table Minimum and maximum values of the time series of hospital admissions, temperature and air pollutants at each location [min, max]. (PDF)

S3 Table Mean and standard deviation (sd) of the time series of hospital admissions, temperature and air pollutants at each location (mean ± sd). (PDF)

S4 Table Indication of available/selected covariates, (*p*, *q*) model orders and cluster belonging for M_F_ models. Signage: covariate available and selected (*✓*), covariate available and not selected (X), covariate not available (empty cell).
(PDF)
