# Supplementary material for: Association between respiratory hospital admissions and air quality in Portugal: A count time series approach
Source: PLoS One. 2021 Jul 9;16(7):e0253455. doi: 10.1371/journal.pone.0253455 (PMC8270143; doi:10.1371/journal.pone.0253455)
Supplement: S1 Table — (PDF) [file pone.0253455.s003.pdf]

Station	Abbreviation	Time Period	Lat	Lon	Environment	Influence
Minho	MINH	2006-2011	41.80	-8.69	R	B
Frossos	FROS	2005-2011	41.57	-8.46	SU	B
Fr. Bartolomeu	FBAR	2005-2011	41.55	-8.41	U	T
Cónego	CONE	2005-2011	41.45	-8.30	U	T
Douro Norte	DOUR	2010-2017	41.37	-7.79	R	B
Burgães	BURG	2010-2016	41.35	-8.46	U	B
Mindelo	MIND	2010-2016	41.31	-8.72	SU	B
Paços Ferreira	PACO	2010-2016	41.27	-8.38	U	B
VNTelha	VNTE	2005-2011	41.25	-8.66	SU	B
Vermoim	VERM	2005-2011	41.24	-8.62	U	T
Meco-Perafita	MECO	2005-2011	41.23	-8.71	SU	I
Leça	LECA	2005-2011	41.22	-8.63	SU	B
Pe Moreira	PEMO	2010-2014	41.21	-8.34	U	T
Valongo	VALO	2009-2014	41.21	-8.55	U	B
Custóias	CUST	2005-2011	41.20	-8.64	SU	B
Sr.ª Hora	SHORA	2005-2011	41.19	-8.66	U	T
Fr. Sá Carneiro	FSAC	2009-2014	41.16	-8.59	U	T
Sobreiras	SOBR	2010-2014	41.15	-8.66	U	B
Estarreja	ESTA	2005-2017	40.76	-8.57	SU	B
Fornelo Monte	FMON	2005-2013	40.64	-8.10	R	B
Aveiro	AVEI	2005-2017	40.64	-8.65	U	T
Ílhavo	ILHA	2005-2017	40.59	-8.67	SU	B
Fundão	FUND	2010-2017	40.23	-7.30	R	B
Av. F. Magalhães	AVRFM	2009-2017	40.21	-8.40	U	T
Instituto Geofísico	INST	2009-2015	40.21	-8.41	U	B
Montemor-o-Velho	MONT	2008-2015	40.18	-8.68	R	B
Ervedeira	ERVE	2008-2017	39.92	-8.89	R	B
Chamusca	CHAM	2005-2017	39.35	-8.47	R	B
Lourinhã	LOURI	2009-2013	39.28	-9.25	R	B
Alverca	ALV	2009-2017	38.90	-9.04	U	B
Loures	LOUR	2005-2011	38.83	-9.16	U	B
Odivelas	ODIV	2005-2017	38.80	-9.18	U	T
Mem Martins	MEM	2011-2017	38.79	-9.35	U	B
Olivais	OLIV	2005-2017	38.77	-9.11	U	B
Reboleira	REBO	2011-2017	38.75	-9.23	U	B
Entrecampos	ENTRE	2005-2017	38.75	-9.15	U	T
St.ª Cruz Benfica	CRUZ	2011-2017	38.75	-9.20	U	T
Alfragide	ALF	2011-2017	38.74	-9.21	U	B
Beato	BEAT	2005-2011	38.73	-9.11	U	B
Av. Liberdade	ALIB	2005-2013	38.72	-9.15	U	T
Restelo	REST	2011-2017	38.71	-9.21	U	B
Quinta Marquês	QUINT	2011-2017	38.70	-9.32	U	B
Lavradio	LAVR	2012-2017	38.67	-9.05	U	I
Laranjeiro	LARAN	2011-2017	38.66	-9.16	U	B
Escavadeira	ESCA	2005-2011	38.66	-9.07	U	I
Fernando Pó	FPO	2007-2017	38.64	-8.69	R	B
Paio Pires	PAIO	2007-2011	38.62	-9.08	SU	I
Terena	TERE	2010-2017	38.62	-7.40	R	B
Arcos	ARCS	2009-2017	38.53	-8.89	U	B
Quebedo	QUEB	2005-2017	38.52	-8.89	U	T
Monte Velho	VELHO	2009-2017	38.08	-8.80	R	B
Santiago do Cacém	SANT	2011-2017	38.02	-8.70	U	I
Monte Chãos	CHAOS	2005-2017	37.95	-8.84	SU	I
Sonega	SONE	2005-2017	37.87	-8.72	R	I
Cerro	CERR	2005-2017	37.31	-7.68	R	B
David Neto	DAVI	2005-2017	37.14	-8.54	U	T
Malpique	MALP	2005-2017	37.09	-8.25	U	B
Joaquim Magalhães	JOAQ	2005-2017	37.02	-7.93	U	B
