# Supplementary material for: Association between respiratory hospital admissions and air quality in Portugal: A count time series approach
Source: PLoS One. 2021 Jul 9;16(7):e0253455. doi: 10.1371/journal.pone.0253455 (PMC8270143; doi:10.1371/journal.pone.0253455)
Supplement: S2 Table — (PDF) [file pone.0253455.s004.pdf]

Station	# Hospital Admissions	Temp (ºC)	$PM_{2.5}$ ($\mu g/m^3$)	PM_{10} ($\mu g/m^3$)	NO_x ($\mu g/m^3$)	NO_2 ($\mu g/m^3$)	O_3 ($\mu g/m^3$)	SO_2 ($\mu g/m^3$)	CO (mg/m^3)
**MINH**	[0, 19]	[3.9, 39.0]	[1.0, 198.0]	[2.0, 184.0]	[0.8, 120.2]	[0.3, 102.1]	[33.0, 216.0]	[0.0, 138.0]	
**FROS**	[0, 46]	[3.0, 39.4]		[0.0, 388.0]	[1.1, 597.2]	[0.2, 121.0]	[3.0, 239.0]	[0.0, 101.0]	
**FBAR**	[1, 46]	[3.0, 39.4]		[9.0, 1812.0]	[11.6, 1442.3]	[6.1, 297.7]			[0.2, 7.7]
**CONE**	[1, 46]	[3.0, 39.4]		[7.0, 442.0]	[0.0, 907.1]	[0.0, 197.7]			[0.2, 5.2]
**DOUR**	[0, 19]	[0.6, 40.5]	[0.0, 107.0]	[2.0, 482.0]	[0.0, 104.7]	[0.0, 94.6]	[43.0, 353.0]	[0.0, 53.0]	
**BURG**	[0, 31]	[4.8, 38.6]		[0.0, 425.0]	[5.6, 378.6]	[4.4, 118.7]	[4.4, 133.0]		
**MIND**	[3, 62]	[4.8, 38.6]		[3.0, 511.0]	[2.4, 422.6]	[1.3, 138.0]	[13.0, 188.0]		
**PACO**	[3, 69]	[2.9, 38.6]	[0.0, 345.0]	[0.0, 562.0]	[3.3, 355.6]	[1.0, 204.1]	[8.0, 215.0]		
**VNTE**	[6, 81]	[3.3, 38.0]		[4.0, 531.0]	[0.0, 866.4]	[0.0, 223.2]	[4.0, 234.0]	[0.0, 341.0	[0.0, 24.2]
**VERM**	[8, 86]	[3.3, 38.0]	[0.0, 319.0]	[2.0, 337.0]	[3.0, 919.2]	[1.0, 221.7]	[5.0, 238.0]	[0.0, 283.0]	[0.1, 5.7]
**MECO**	[6, 77]	[3.3, 38.0]		[0.0, 425.0]	[2.4, 523.7]	[2.1, 174.0]	[6.0, 216.0]	[0.0, 323.0]	[0.1, 12.4]
**LECA**	[7, 83]	[3.3, 38.0]		[4.0, 373.0]	[1.9, 754.7]	[1.8, 373.0]	[2.0, 229.0]	[0.0, 268.0]	[0.2, 3.7]
**PEMO**	[2, 54]	[2.9, 37.9]		[6.0, 327.0]	[10.9, 574.0]	[6.9, 152.3]			
**VALO**	[5, 96]	[4.8, 40.7]		[4.0, 279.0]	[9.6, 964.1]	[7.0, 408.8]	[2.0, 256.0]		
**CUST**	[7, 82]	[3.3, 38.0]		[5.0, 355.0]	[3.9, 1085.6]	[2.0, 313.9]	[8.0, 211.0]	[0.0, 452.0]	
**SHORA**	[7, 80]	[3.3, 38.0]		[2.0, 769.0]	[8.1, 847.5]	[3.9, 300.0]		[0.0, 361.0]	[0.1, 4.8]
**FSAC**	[5, 93]	[4.8, 40.7]		[9.0, 520.0]	[28.9, 972.2]	[7.4, 268.9]			[0.1, 7.9]
**SOBR**	[5, 86]	[8.1, 42.8]	[0.0, 64.0]	[0.0, 293.0]	[5.7, 538.4]	[4.3, 228.7]	[17.0, 197.0]		
**ESTA**	[2, 57]	[7.7, 39.3]	[1.0, 258.0]	[4.0, 311.0]	[5.0, 449.0]	[1.0, 180.0]	[0.0, 332.0]	[0.0, 509.0]	
**FMON**	[0, 22]	[0.8, 38.9]		[0.0, 337.0]	[0.0, 195.0]	[0.0, 92.0]	[11.0, 256.0]	[0.0, 66.0]	
**AVEI**	[0, 29	[7.7, 39.3]		[8.0, 378.0]	[3.0, 1438.0]	[9.0, 343.0]			[0.1, 8.5]
**ILHA**	[0, 27]	[7.7, 39.3]		[2.0, 237.0]	[0.0, 247.0]	[0.0, 125.0]	[18.0, 267.0]	[0.0, 131.0]	
**FUND**	[0, 19]	[1.3, 40.7]	[1.0, 196.0]	[1.0, 220.0]	[0.0, 70.0]	[0.0, 41.0]	[21.0, 210.0]	[0.0, 22.0]	
**AVFRM**	[0, 40]	[8.3, 41.2]		[7.0, 214.0]	[0.0, 1179.0]	[0.0, 334.0]			[0.1, 2.6]
**INST**	[0, 40]	[8.3, 40.8]		[1.0, 178.0]	[0.0, 306.0]	[1.0, 119.0]	[7.0, 217.0]	[0.0, 57.0]	
**MONT**	[0, 34]	[7.7, 39.0]		[6.0, 161.0]	[0.0, 95.0]	[0.0, 229.0]	[8.0, 212.0]	[0.0, 94.0]	
**ERVE**	[0, 27]	[5.8, 41.6]	[0.0, 120.0]	[3.0, 1000.0]	[0.0, 66.0]	[0.0, 87.0]	[25.0, 231.0]	[0.0, 184.0]	
**CHAM**	[0, 18]	[5.0, 44.7]	[1.0, 306.0]	[4.4, 440.7]	[2.6, 170.3]	[1.7, 74.1]	[25.8, 283.6]		
**LOURI**	[0, 19]	[7.6, 33.4]		[4.2, 86.6]	[2.3, 139.2]	[2.2, 58.7]	[28.4, 232.7]		
**ALV**	[2, 138]	[7.3, 40.8]		[2.2, 279.6]	[5.5, 886.6]	[3.9, 151.4]	[3.9, 151.4]	[0.0, 400.3]	
**LOUR**	[12, 123]	[7.0, 40.6]		[8.9, 465.1]	[2.5, 970.0]	[4.8, 168.2]	[5.0, 248.3]	[0.0, 97.3]	[0.1, 3.7]
**ODIV**	[5, 157]	[7.0, 40.8]		[4.7, 402.4]	[13.7, 1087.2]	[7.4, 319.4]	[6.5, 244.6]		[0.1, 3.6]
**MEM**	[5, 135]	[7.5, 48.6]	[2.0, 78.5]	[2.1, 125.7]	[3.2, 469.8]	[2.3, 153.8]	[17.2, 235.9]	[0.0, 13.1]	
**OLIV**	[6, 158]	[7.0, 40.8]	[2.0, 324.0]	[4.4, 190.9]	[9.6, 1924.4]	[7.8, 430.3]	[4.5, 223.8]	[0.0, 145.7]	[0.1, 5.1]
**REBO**	[8, 159]	[7.5, 48.6]		[3.3, 152.1]	[5.5, 766.3]	[5.4, 175.7]	[3.2, 189.6]		
**ENTRE**	[5, 158]	[7.0, 40.8]	[4.0, 423.8]	[7.1, 558.1]	[14.0, 1480.6]	[11.8, 296.4]	[6.5, 189.3]	[0.0, 125.7]	[0.1, 4.8]
**CRUZ**	[9, 164]	[7.5, 48.6]		[12.1, 276.5]	[22.0, 1190.7]	[10.8, 222.1]			[0.2, 4.3]
**ALF**	[7, 160]	[7.5, 48.6]			[0.0, 1110.0]	[1.2, 343.7]	[5.8, 218.1]	[0.0, 26.3]	[0.0, 15.5]
**BEAT**	[8, 96]	[7.0, 40.6]			[7.7, 854.9]	[7.7, 235.7]	[9.8, 201.6]	[0.0, 195.6]	[0.1, 3.4]
**ALIB**	[14, 153]	[7.5, 39.4]		[13.0, 480.3]	[0.0, 1744.2]	[20.1, 472.9]			[0.2, 5.4]
**REST**	[9, 162]	[8.2, 41.4]		[10.0, 190.7]	[4.1, 369.4]	[3.2, 204.2]	[9.2, 232.1]		
**QUINT**	[6, 136]	[7.5, 48.6]		[2.2, 158.7]	[0.0, 417.9]	[3.4, 143.8]	[14.3, 215.9]		
**LAVR**	[4, 115]	[7.2, 40.5]		[3.1, 186.5]	[6.9, 489.8]	[5.5, 136.3]		[0.0, 245.2]	
**LARAN**	[5, 131]	[8.2, 41.4]	[3.0, 109.2]	[4.4, 179.8]	[6.6, 1064.3]	[5.4, 216.6]	[13.6, 211.3]		[0.1, 3.6]
**ESCA**	[11, 110]	[7.0, 39.7]		[6.7, 400.7]	[6.0, 782.0]	[4.9, 437.8]	[8.0, 232.5]	[0.0, 1104.3]	[0.1, 3.7]
**FPO**	[0, 13]	[5.3. 44.1]	[1.0, 109.0]	[2.2, 174.3]	[3.1, 196.9]	[2.7, 57.4]	[2.7, 57.4]	[0.0, 79.8]	
**PAIO**	[8, 83]	[7.0, 39.7]		[14.9, 255.6]	[11.4, 656.0]	[6.7, 150.6]	[7.1, 207.2]	[0.0, 422.1]	[0.1, 4.2]
**TERE**	[0, 6]	[4.3, 45.8]	[0.0, 2060.0]	[5.0, 442.0]	[0.0, 73.0]	[0.0, 58.0]	[10.0, 147.0]	[1.0, 20.0]	
**ARCS**	[0, 29]	[7.2, 43.0]		[9.5, 312.8]	[5.7, 377.5]	[4.5, 135.2]	[20.9, 225.0]		[0.1, 1.8]
**QUEB**	[0, 29]	[7.2, 43.0]		[4.7, 206.5]	[7.4, 981.8]	[6.2, 186.8]		[0.0, 502.7]	[0.1, 4.2]
**VELHO**	[0, 7]	[6.5, 40.7]	[7.0, 181.0]	[13.0, 408.0]	[0.0, 56.0]	[0.0, 41.0]		[0.0, 131.0]	[0.1, 1.2]
**SANT**	[0, 8]	[6.5, 40.7]		[4.0, 470.0]	[0.0, 145.0]	[0.0, 90.0]	[28.0, 188.0]	[0.0, 284.0]	[0.1, 0.7]
**CHAOS**	[0, 7]	[6.5, 40.7]			[0.0, 175.0]	[0.0, 112.0]	[26.0, 218.0]	[0.0, 599.0]	
**SONE**	[0, 9]	[6.5, 40.7]			[0.0, 311.0]	[0.0, 154.0]	[16.0, 220.0]	[0.0, 417.0]	
**CERR**	[0, 6]	[5.0, 43.6]	[1.0, 138.0]	[0.0, 343.4]	[0.0, 281.7]	[0.0, 64.4]	[36.0, 182.7]	[0.0, 162.0]	
**DAVI**	[0, 14]	[8.5, 41.0]		[8.9, 337.5]	[3.9, 840.3]	[0.1, 249.0]			[0.0, 5.1]
**MALP**	[0, 14]	[9.0, 37.8]		[7.6, 273.9]	[2.3, 566.0]	[0.0, 128.8]	[21.8, 196.3]	[0.0, 156.6]	
**JOAQ**	[0, 18]	[9.0, 37.8]	[0.0, 472.0]	[7.8, 296.0]	[1.7, 435.1]	[0.0, 128.3]	[29.9, 207.3]	[0.0, 204.9]	
