# Supplementary material for: Association between respiratory hospital admissions and air quality in Portugal: A count time series approach
Source: PLoS One. 2021 Jul 9;16(7):e0253455. doi: 10.1371/journal.pone.0253455 (PMC8270143; doi:10.1371/journal.pone.0253455)
Supplement: S3 Table — (PDF) [file pone.0253455.s005.pdf]

**Station**	**# Hospital Admissions**	**Temp (ºC)**	**$PM_{2.5}$** ($\mu g/m^3$)	**PM_{10}** ($\mu g/m^3$)	**NO_x** ($\mu g/m^3$)	**NO_2** ($\mu g/m^3$)	**O_3** ($\mu g/m^3$)	**SO_2** ($\mu g/m^3$)	**CO** (mg/m^3)
**MINH**	6.4 ± 3.5	21.4 ± 6.5	13.9 ± 10.5	20.8 ± 13.0	8.7 ± 6.0	7.1 ± 5.3	87.6 ± 26.1	4.5 ± 9.5	
**FROS**	15.1 ± 7.0	21.4 ± 6.6		49.6 ± 33.2	78.5 ± 74.7	36.1 ± 18.0	77.8 ± 30.0	6.0 ± 6.0	
**FBAR**	16.2 ± 7.3	21.4 ± 6.6		71.1 ± 69.2	319.9 ± 211.4	82.2 ± 34.3			1.2 ± 0.8
**CONE**	17.5 ± 7.0	21.4 ± 6.6		62.6 ± 35.4	211.6 ± 129.5	74.9 ± 26.1			1.1 ± 0.7
**DOUR**	3.5 ± 2.4	20.8 ± 8.4	8.2 ± 7.6	24.5 ± 21.2	9.1 ± 5.3	7.0 ± 4.7	101.3 ± 30.0	4.1 ± 4.6	
**BURG**	11.5 ± 5.2	19.5 ± 5.2		44.0 ± 31.8	67.5 ± 53.7	36.8 ± 17.1	38.1 ± 18.6		
**MIND**	23.3 ± 9.2	19.5 ± 5.2		49.1 ± 30.6	66.6 ± 57.9	39.1 ± 23.0	69.6 ± 21.5		
**PACO**	23.7 ± 9.3	19.5 ± 7.0	17.2 ± 19.1	33.9 ± 24.9	65.2 ± 52.0	35.1 ± 22.9	67.1 ± 27.1		
**VNTE**	31.2 ± 11.2	19.6 ± 5.3		65.5 ± 39.6	85.1 ± 71.2	46.1 ± 24.3	76.1 ± 26.4	13.1 ± 20.0	0.7 ± 0.9
**VERM**	33.5 ± 11.9	19.6 ± 5.3	41.3 ± 40.0	75.6 ± 48.7	147.5 ± 131.9	66.2 ± 32.6	76.9 ± 27.7	18.1 ± 20.0	0.9 ± 0.7
**MECO**	29.6 ± 10.7	19.6 ± 5.3		71.4 ± 37.1	85.2 ± 69.9	46.9 ± 27.3	79.2 ± 25.1	12.1 ± 24.5	0.6 ± 0.4
**LECA**	32.0 ± 11.4	19.6 ± 5.3		70.1 ± 43.8	134.3 ± 115.6	64.8 ± 42.1	71.3 ± 27.6	13.4 ± 20.7	0.9 ± 0.5
**PEMO**	18.2 ± 7.4	19.5 ± 7.1		43.3 ± 29.7	111.7 ± 81.4	54.2 ± 24.4			
**VALO**	37.6 ± 13.5	21.5 ± 6.0		46.3 ± 26.4	137.9 ± 119.7	68.7 ± 40.2	73.9 ± 25.7		
**CUST**	32.0 ± 11.4	19.6 ± 5.3		77.8 ± 45.9	120.6 ± 115.1	59.0 ± 41.1	75.2 ± 23.6	24.2 ± 31.0	
**SHORA**	31.9 ± 11.4	19.6 ± 5.3		63.8 ± 45.4	161.5 ± 110.0	77.4 ± 35.4		35.4 ± 48.6	0.9 ± 0.6
**FSAC**	37.0 ± 13.1	21.5 ± 6.0		50.9 ± 33.3	259.2 ± 136.4	96.2 ± 38.6			1.0 ± 0.6
**SOBR**	35.2 ± 12.7	21.8 ± 5.9	11.2 ± 8.7	54.8 ± 32.6	98.3 ± 77.1	58.7 ± 29.8	75.2 ± 22.3		
**ESTA**	19.0 ± 7.9	19.8 ± 4.7	35.6 ± 29.4	55.2 ± 35.9	84.7 ± 65.4	40.9 ± 22.9	81.4 ± 30.3	19.0 ± 23.3	
**FMON**	6.3 ± 3.3	18.6 ± 7.8		23.9 ± 19.5	8.7 ± 8.4	7.2 ± 5.9	95.7 ± 28.9	7.5 ± 5.4	
**AVEI**	9.7 ± 4.6	19.8 ± 4.7		57.2 ± 38.3	112.6 ± 112.7	56.8 ± 33.3			0.7 ± 0.6
**ILHA**	9.4 ± 4.5	19.8 ± 4.7		40.2 ± 26.6	27.3 ± 23.1	21.6 ± 14.5	82.0 ± 26.0	5.0 ± 6.9	
**FUND**	3.1 ± 2.2	21.6 ± 8.6	11.5 ± 10.1	22.6 ± 16.8	9.5 ± 6.8	10.2 ± 5.1	92.2 ± 20.9	1.6 ± 1.5	
**AVFRM**	10.1 ± 6.2	22.4 ± 6.5		45.0 ± 21.1	177.4 ± 127.3	61.8 ± 28.0			0.6 ± 0.3
**INST**	11.3 ± 5.9	22.2 ± 6.5		40.0 ± 19.6	50.3 ± 40.7	34.3 ± 20.7	78.6 ± 26.3	3.4 ± 3.8	
**MONT**	9.4 ± 5.0	20.6 ± 5.2		33.5 ± 16.9	17.3 ± 13.3	22.6 ± 19.4	85.5 ± 27.1	6.9 ± 9.0	
**ERVE**	6.0 ± 3.4	21.1 ± 5.7	19.7 ± 15.7	31.7 ± 26.4	12.7 ± 8.4	13.5 ± 10.9	82.7 ± 24.1	15.4 ± 15.2	
**CHAM**	3.9 ± 2.5	23.2 ± 7.6	15.6 ± 12.4	27.0 ± 17.7	14.0 ± 8.1	11.7 ± 6.2	95.3 ± 26.8		
**LOURI**	6.7 ± 3.4	18.1 ± 3.4		26.0 ± 11.2	15.7 ± 13.9	13.0 ± 9.4	90.0 ± 23.6		
**ALV**	28.4 ± 12.5	21.9 ± 6.4		32.8 ± 16.0	90.3 ± 84.5	44.6 ± 25.1	44.8 ± 25.1	3.2 ± 7.8	
**LOUR**	51.6 ± 18.6	21.7 ± 6.4		43.8 ± 22.1	113.3 ± 128.0	49.2 ± 26.2	82.8 ± 26.7	4.4 ± 6.5	0.5 ± 0.3
**ODIV**	57.3 ± 22.5	21.8 ± 6.3		41.3 ± 22.5	147.0 ± 130.1	61.2 ± 33.1	79.0 ± 23.8		0.5 ± 0.4
**MEM**	54.0 ± 22.3	22.3 ± 5.8	15.9 ± 9.4	30.1 ± 14.1	50.0 ± 58.9	31.9 ± 25.7	86.6 ± 22.5	0.9 ± 1.4	
**OLIV**	56.8 ± 22.2	21.8 ± 6.3	22.5 ± 14.4	40.9 ± 21.8	175.0 ± 187.4	70.9 ± 41.0	79.5 ± 25.4	3.2 ± 5.3	0.6 ± 0.5
**REBO**	62.2 ± 25.5	22.3 ± 5.8		29.6 ± 15.7	83.7 ± 89.2	45.3 ± 30.4	81.8 ± 24.0		
**ENTRE**	57.3 ± 22.4	21.8 ± 6.3	25.3 ± 16.1	47.6 ± 28.1	219.2 ± 196.5	84.0 ± 40.4	71.8 ± 24.1	5.4 ± 7.2	0.7 ± 0.5
**CRUZ**	65.9 ± 26.9	22.3 ± 5.8		56.2 ± 29.7	226.0 ± 200.2	72.2 ± 36.2			0.8 ± 0.5
**ALF**	61.0 ± 25.2	22.3 ± 5.8			158.1 ± 158.5	70.2 ± 47.7	72.5 ± 22.4	1.9 ± 1.9	0.7 ± 0.8
**BEAT**	39.5 ± 14.9	21.7 ± 6.4			106.1 ± 104.8	58.7 ± 30.9	80.3 ± 24.2	4.0 ± 6.7	0.4 ± 0.4
**ALIB**	59.4 ± 21.4	22.0 ± 6.0		67.9 ± 32.4	334.7 ± 210.5	114.3 ± 47.9			0.9 ± 0.6
**REST**	65.9 ± 26.7	22.5 ± 6.0		37.4 ± 17.3	74.3 ± 62.5	48.3 ± 26.9	84.6 ± 25.1		
**QUINT**	55.9 ± 22.9	22.3 ± 5.8		29.3 ± 13.9	42.1 ± 49.0	34.9 ± 25.8	84.2 ± 23.2		
**LAVR**	46.0 ± 19.9	22.1 ± 6.0		33.8 ± 18.8	56.2 ± 51.1	36.0 ± 20.7		3.8 ± 9.2	
**LARAN**	53.8 ± 2.2	22.5 ± 6.0	21.0 ± 13.0	36.0 ± 19.2	119.9 ± 138.5	58.4 ± 34.5	85.7 ± 25.8		0.5 ± 0.4
**ESCA**	44.4 ± 16.1	22.1 ± 6.1		54.0 ± 33.8	132.7 ± 98.4	54.8 ± 27.0	87.6 ± 28.0	31.7 ± 50.2	0.5 ± 0.5
**FPO**	3.1 ± 2.3	24.1 ± 7.2	17.2 ± 11.1	31.2 ± 17.3	19.2 ± 11.6	15.1 ± 7.8	15.1 ± 7.8	2.0 ± 3.1	
**PAIO**	37.5 ± 12.9	22.0 ± 5.9		65.3 ± 35.5	98.7 ± 78.2	52.3 ± 24.8	82.7 ± 26.9	7.9 ± 23.0	0.7 ± 0.5
**TERE**	0.6 ± 0.8	24.2 ± 8.6	30.9 ± 43.4	50.0 ± 34.3	12.1 ± 7.8	8.6 ± 5.4	70.2 ± 18.7	2.7 ± 1.5	
**ARCS**	8.2 ± 4.7	23.3 ± 6.6		40.1 ± 22.1	54.8 ± 49.1	34.5 ± 20.1	89.9 ± 24.8		0.4 ± 0.2
**QUEB**	8.2 ± 4.6	23.2 ± 6.6		44.6 ± 22.1	105.2 ± 100.7	49.0 ± 25.8		3.4 ± 11.7	0.5 ± 0.4
**VELHO**	0.8 ± 1.0	20.5 ± 4.7	29.2 ± 16.6	50.7 ± 31.6	10.0 ± 6.4	7.0 ± 4.9		5.7 ± 7.7	0.2 ± 0.1
**SANT**	1.1 ± 1.2	20.5 ± 4.7		74.3 ± 52.7	9.0 ± 10.1	6.2 ± 6.4	86.8 ± 22.4	8.6 ± 18.9	0.3 ± 0.1
**CHAOS**	1.0 ± 1.1	20.4 ± 4.7			15.0 ± 12.8	11.8 ± 10.4	86.8 ± 22.6	17.7 ± 34.7	
**SONE**	1.0 ± 1.2	20.4 ± 4.7			10.7 ± 9.6	8.6 ± 6.9	78.2 ± 27.7	20.6 ± 34.6	
**CERR**	0.9 ± 1.0	23.2 ± 7.9	12.4 ± 8.2	27.4 ± 20.8	6.2 ± 11.0	5.2 ± 4.0	96.9 ± 19.2	6.9 ± 13.1	
**DAVI**	3.4 ± 2.5	23.6 ± 5.9		56.6 ± 27.5	98.3 ± 72.0	46.6 ± 21.8			0.9 ± 0.6
**MALP**	3.0 ± 2.1	22.5 ± 5.4		40.8 ± 19.0	42.0 ± 37.3	30.5 ± 19.9	92.8 ± 19.9	12.1 ± 9.8	
**JOAQ**	3.8 ± 2.5	22.5 ± 5.4	21.2 ± 29.8	37.7 ± 16.8	40.5 ± 35.6	31.9 ± 21.4	90.9 ± 20.4	12.9 ± 17.6	
