# Supplementary material for: Association between respiratory hospital admissions and air quality in Portugal: A count time series approach
Source: PLoS One. 2021 Jul 9;16(7):e0253455. doi: 10.1371/journal.pone.0253455 (PMC8270143; doi:10.1371/journal.pone.0253455)
Supplement: S4 Table — Signage: covariate available and selected (✓), covariate available and not selected (X), covariate not available (empty cell). (PDF) [file pone.0253455.s006.pdf]

Station	Temp	$PM_{2.5}$	PM_{10}	NO_x	NO_2	O_3	SO_2	CO	p	q	Cluster
**MINH**	✓	X	✓	X	✓	X	✓		7	7	C2
**FROS**	✓		X	✓	X	X	X		7	7	C2
**FBAR**	✓		X	✓	X			✓	7	7	C2
**CONE**	✓		X	X	✓			X	7	7	C2
**DOUR**	✓	X	X	✓	X	X	X		7	7	C2
**BURG**	✓		X	✓	X	X			7	7	C2
**MIND**	✓		✓	✓	X	X			7	7	C2
**PACO**	✓	✓	X	✓	X	X			7	7	C2
**VNTE**	✓		✓	X	✓	X	X	X	7	7	C2
**VERM**	✓	X	✓	✓	X	✓	X	X	7	7	C2
**MECO**	✓		✓	X	X	✓	X	X	7	7	C2
**LECA**	✓		X	X	X	✓	X	✓	7	7	C2
**PEMO**	✓		✓	X	✓				7	7	C2
**VALO**	✓		✓	X	✓	✓			7	7	C2
**CUST**	✓		✓	X	X	✓	X		7	7	C2
**SHORA**	✓		✓	X	✓		X	X	7	7	C2
**FSAC**	✓		X	✓	X			✓	7	7	C2
**SOBR**	✓	X	✓	X	✓	✓			7	7	C2
**ESTA**	✓	✓	X	X	✓	✓	X		6	6	C2
**FMON**	✓		✓	✓	X	✓	✓		7	7	C2
**AVEI**	✓		✓	✓	X			X	7	7	C2
**ILHA**	✓		✓	✓	X	✓	X		7	7	C2
**FUND**	✓	X	✓	X	X	✓	X		7	7	C2
**AVRFM**	✓		X	X	X			X	6	5	C2
**INST**	✓		X	X	X	X	✓		7	7	C2
**MONT**	X		X	X	X	X	X		7	7	C2
**ERVE**	✓	X	X	✓	X	✓	X		7	7	C2
**CHAM**	✓	X	✓	✓	X	X			7	7	C2
**LOURI**	✓		✓	✓	X	✓			7	7	C2
**ALV**	✓		✓	X	✓	X	X		7	7	C2
**LOUR**	✓		✓	X	X	X	X	X	7	7	C2
**ODIV**	✓		X	X	X	X		X	7	7	C1
**MEM**	X	X	✓	X	✓	✓	X		7	7	C1
**OLIV**	✓	X	X	X	X	✓	X	X	7	7	C1
**REBO**	✓		✓	X	X	✓			7	7	C1
**ENTRE**	✓	X	✓	✓	X	X	X	X	7	7	C1
**CRUZ**	✓		X	X	✓			✓	7	7	C1
**ALF**	✓			X	X	X	X	X	7	7	C1
**BEAT**	✓			X	X	X	✓	X	7	7	C2
**ALIB**	✓		✓	✓	X			X	6	6	C1
**REST**	✓		X	X	✓	X			7	7	C1
QUINT	X		X	X	X	✓			7	7	C1
**LAVR**	✓		✓	X	X		X		7	7	C1
**LARAN**	✓	X	X	✓	X	X		✓	7	7	C1
**ESCA**	✓		X	X	✓	X	X	✓	7	7	C2
**FPO**	✓	✓	X	X	✓	X	✓		7	7	C2
**PAIO**	✓		✓	✓	X	✓	X	X	6	6	C2
**TERE**	✓	✓	X	X	X	X	X		5	7	C2
**ARCS**	✓		✓	X	X	X		X	7	7	C2
**QUEB**	✓		X	✓	X		✓	✓	7	7	C2
**VELHO**	✓	✓	X	X	X		X	X	7	7	C2
**SANT**	✓		X	✓	X	✓	X	✓	7	7	C2
**CHAOS**	✓			X	X	X	X		5	5	C2
**SONE**	✓			X	✓	✓	✓		7	7	C2
**CERR**	✓	X	X	X	✓	X	X		6	7	C2
**DAVI**	✓		✓	✓	X			✓	7	7	C2
**MALP**	✓		✓	X	X	✓	✓		7	7	C2
**JOAQ**	✓	X	✓	X	X	X	✓		7	7	C2
